# Genomic characterization and probiotic potential assessment of an exopolysaccharide-producing strain *Pediococcus pentosaceus* LL-07 isolated from fermented meat

**DOI:** 10.1186/s12866-024-03304-6

**Published:** 2024-04-25

**Authors:** Kuan Lu, Xueya Wang, Ying Zhou, Qiujin Zhu

**Affiliations:** 1 Key Laboratory of Plant Resource Conservation and Germplasm Innovation in Mountainous Region (Ministry of Education), College of Life Sciences/Institute of Agro-Bioengineering, Guizhou University, Guizhou Province, Guiyang, 550025 China; 2 Guizhou Province Key Laboratory of Agricultural and Animal Products Storage and Processing, School of Liquor and Food Engineering, Guizhou University, Guizhou, Guiyang, 550025 China; 3 Chili Pepper Research Institute, Guizhou Provincial Academy of Agricultural Sciences, Guizhou, Guiyang, 550006 China

**Keywords:** *Pediococcus pentosaceus*, Whole-genome sequencing, Exopolysaccharide, Comparative genomics

## Abstract

**Background:**

The genomic information available for *Pediococcus pentosaceus* is primarily derived from fermented fruits and vegetables, with less information available from fermented meat. *P. pentosaceus* LL-07, a strain isolated from fermented meat, has the capability of producing exopolysaccharides (EPS). To assess the probiotic attributes of *P. pentosaceus* LL-07, we conducted whole-genome sequencing (WGS) using the PacBio SequelIIe and Illumina MiSeq platforms, followed by in vitro experiments to explore its probiotic potential.

**Results:**

The genome size of *P. pentosaceus* LL-07 is 1,782,685 bp, comprising a circular chromosome and a circular plasmid. Our investigation revealed the absence of a CRISPR/Cas system. Sugar fermentation experiments demonstrated the characteristics of carbohydrate metabolism. *P. pentosaceus* LL-07 contains an EPS synthesis gene cluster consisting of 13 genes, which is different from the currently known gene cluster structure. NO genes associated with hemolysis or toxin synthesis were detected. Additionally, eighty-six genes related to antibiotic resistance were identified but not present in the prophage, transposon or plasmid. In vitro experiments demonstrated that *P. pentosaceus* LL-07 was comparable to the reference strain *P. pentosaceus* ATCC25745 in terms of tolerance to artificial digestive juice and bile, autoaggregation and antioxidation, and provided corresponding genomic evidence.

**Conclusion:**

This study confirmed the safety and probiotic properties of *P. pentosaceus* LL-07 via complete genome and phenotype analysis, supporting its characterization as a potential probiotic candidate.

**Supplementary Information:**

The online version contains supplementary material available at 10.1186/s12866-024-03304-6.

## Background

Probiotics have received considerable attention due to their positive impact on human health. They can improve the intestinal environment, enhance immunity, nutritional status, and prevent chronic diseases [1]. Probiotics have a wide range of applications in food, agriculture, and pharmaceutical industries. In order to play a beneficial role, probiotics need to colonize the intestinal tract in a live form. Therefore, candidate probiotic strains must possess specific characteristics, including digestive fluid and bile salt tolerance, adhesion, and safety [2].

*Pediococcus pentosaceus*, a type of lactic acid bacteria (LAB), is a generally recognized as safe (GRAS) strain. In addition to the acid-producing function of LAB, *P. pentosaceus* is known to significantly enhance the flavor, quality, and safety of fermented products [3–6]. Research indicates that *P. pentosaceus* and its bacteriocins are closely linked to human gastrointestinal tract health (GIT) [7, 8]. Experimental evidence suggests that *P. pentosaceus* has potential as a probiotic, in addition to its use as a food starter and biological preservative [9–11].

Exopolysaccharides (EPS) are secondary metabolites produced by microorganisms during their growth and metabolism. EPS has a variety of physiological functions [12] and plays a specific role in improving food texture [13]. Some strains of *P. pentosaceus* produce EPS with active functions during the growth and metabolism, in addition to generating active substances such as bacteriocins [14, 15]. The function of EPS derived from *P. pentosaceus* has been scientifically studied. Researchers are further exploring and confirming these findings, which suggest a potential connection between the probiotic function of *P. pentosaceus* and EPS. [16].

Advances in genome sequencing technology and the increasing availability of sequence analysis tools have made genome characterization of microorganisms possible and essential for defining accurate classification of new strains, useful information, and their safety. [17, 18]. The study of genome sequences enables a rigorous safety assessment, including the determination of antimicrobial resistance and the presence of virulence factors. These aspects are crucial when introducing new strains into food applications [19, 20]. Since the first *P. pentosaceus* strain was sequenced, as of November 2023, 150 data points related to its genome have been published in the NCBI database, with only 30 complete genome sequences. Of these 30 strains, 21 *P. pentosaceus* strains were isolated from fruits and vegetables, while one was each derived from soil, raw milk, shrimp, or *Homo sapiens*. The sources of the remaining five strains have not been determined. In comparison to LAB such as *Lactiplantibacillus plantarum* and *Streptococcus thermophilus*, *P. pentosaceus* has substantial potential for expanding source diversity and genome database coverage. Distinct from the known 30 strains of *P. pentosaceus*, *P. pentosaceus* LL-07 was isolated from fermented meat (Libo, Guizhou, China), and has demonstrated the ability to produce exopolysaccharides (EPS), underscoring its potential as a probiotic contender. A comprehensive analysis of its traits and possible underlying issues is needed for future development and application. This study aimed to perform whole-genome sequencing of *P. pentosaceus* LL-07 to establish its taxonomy through genetic exploration and comparative genomic analysis and to evaluate its beneficial properties, EPS synthesis pathways, and safety factors. In addition, we experimentally evaluated several probiotic properties of *P. pentosaceus* LL-07, such as tolerance to artificial digestive juice and bile, autoaggregation, and antioxidation.

## Materials and methods

### Bacterial strain and culture conditions

*P. pentosaceus* LL-07 was isolated from fermented meat (Libo, Guizhou, China) and characterized as a bacterium capable of producing EPS. It was initially identified as *P. pentosaceus* through the 16S rRNA gene sequencing. *P. pentosaceus* ATCC25745 was used as a reference.

*P. pentosaceus* LL-07 was inoculated in Man Rogosa Sharpe broth (MRS, Shanghai Bio-way Technology Co., Ltd., China) liquid medium with a 2% (v/v) inoculum and incubated at 37 ℃ for 24 h under static conditions. After two to three activation cultures were performed, the culture was set aside.

*Staphylococcus aureus* ATCC6538 was used as a positive control strain for the hemolysis test and inoculated in Luria–Bertani (LB, Shanghai Sangwei Science and Technology Co., Ltd., Shanghai, China) medium at a concentration of 2% (v/v) inoculum and incubated at 37 °C for 12 h.

Additionally, another thirty complete genomes of *P. pentosaceus*, publicly available on the National Center for Biotechnology Information (NCBI) website (https://www.ncbi.nlm.nih.gov/) (released from 1980 to 2023), were used for comparison (Table 1).

**Table 1:** Source information, BioSample Accession and general genome features of 30 *P. pentosaceus* strains

Strain Name	BioSample Accession	Size (Mb)	GC (%)	CDS	Isolation source	Geographic Location	Plasmid
ATCC 25745	SAMN02598525	1.83	37.40	1,755	Plant	United States	0
FDAARGOS_1009	SAMN16357178	1.75	37.39	1,610	Unknown	Germany: Braunschweig	1
ZZ61	SAMN36291298	1.85	37.00	1,777	Raw milk	China: Xinjiang	0
SL001	SAMN11094516	1.92	37.44	1,826	Soil	China: Dadonghai beach, Sanya	1
GDIAS001	SAMN13611447	1.83	37.10	1,763	Tapioca	China: Guangzhou	0
MGB0941	SAMN22222385	1.96	37.39	1,845	kimchi	South Korea	4
MGB0620	SAMN22222353	2.06	37.46	1,947	kimchi	South Korea	5
SRCM102740	SAMN08707615	1.88	37.41	1,765	Soybean paste (Chonggugjang)	South Korea	1
SRCM102738	SAMN08707613	1.88	37.41	1,770	Soybean paste (Chonggugjang)	South Korea	1
SRCM102739	SAMN08707614	1.90	37.37	1,791	Soybean paste (Chonggugjang)	South Korea	2
SMFM2016-WK1	SAMN35689387	1.81	37.30	1,763	white kimchi	South Korea: Seoul	0
MR001	SAMN13612138	1.80	37.20	1,757	Shrimp	Thailand: Songkhla	0
FDAARGOS_1010	SAMN16357179	1.95	37.36	1,855	Unknown	Germany: Braunschweig	5
SMFM2016-NK1	SAMN35689357	1.80	37.30	1,722	Nappa cabbage kimchi	South Korea: Seoul	0
SMFM2016-YK1	SAMN35689378	1.80	37.30	1,692	Young radish water kimchi	South Korea: Seoul	0
FDAARGOS_1011	SAMN16357180	1.79	37.30	1,712	Unknown	Germany: Braunschweig	0
Ca-4	SAMN33368519	1.94	37.36	1,884	Fermented cherry juice	China: Beijing	3
KCCM 40703	SAMN06447729	1.76	37.20	1,692	Sake mash	Japan	0
SRCM100194	SAMN07224387	1.87	37.38	1,803	food	South Korea	2
SRCM102736	SAMN08707611	1.81	37.39	1,725	Soybean paste (Chonggugjang)	South Korea	1
SS1-3	SAMN07551496	1.84	37.28	1,743	Homo sapiens	South Korea: Bundang	2
FDAARGOS_873	SAMN13450403	1.74	37.20	1,673	Unknown	Not applicable	0
wikim20	SAMN04017317	1.83	37.26	1,703	kimchi	South Korea: Gwangju	3
MGB0055	SAMN22220488	1.82	37.27	1,688	kimchi	South Korea	2
JQI-7	SAMN07701459	1.73	37.20	1,662	Fermented dairy	China	0
SRCM102734	SAMN08707609	1.71	37.40	1,607	Soybean paste(Doenjang)	South Korea	0
FDAARGOS_1134	SAMN16357303	1.74	37.41	1,649	Unknown	Germany: Braunschweig	1
SL4	SAMN02603952	1.79	37.30	1,716	kimchi	Korean	7
EN5	SAMN32423453	1.84	37.35	1,751	phyllosphere epiphyte	China: Cangzhou	1
SRCM100892	SAMN07126152	2.00	37.27	1,593	Food	South Korea	6

### Genome sequencing, assembly and annotation

Typical individual *P. pentosaceus* LL-07 colonies were selected, inoculated in 200 mL of MRS liquid medium, and cultured at 37 ℃ until reaching logarithmic growth. Subsequently, centrifugation was carried out at 16.000 × g for 10 min at 4 ℃, the supernatant was discarded, the bacterial cells were collected, and the medium components were rinsed away with sterile water. Genomic DNA extraction followed the protocols of the bacterial DNA extraction kit (magnetic beads) from Majorbio (Shanghai, China).

Whole-genome sequencing of *P. pentosaceus* LL-07 was conducted by Majorbio BioTech Co. genomic services (Shanghai, China) using PacBio Sequel IIe (Pacbio, America) and NovaSeq6000 (Illumina, America).

Coding sequences (CDS) in the genome were predicted using Glimmer [21] (http://ccb.jhu.edu/software/glimmer/index.shtml). The *P. pentosaceus* LL-07 genome map was predicted using CGView (http://stothard.afns.ualberta.ca/cgview_server/) [22]. tRNAs in the genome were predicted using tRNAscan-SE v2.0 [23] software (http://trna.ucsc.edu/software/). rRNAs contained in the genome were predicted using Barrnap software (https://github.com/tseemann/barrnap). Predictive annotation of possible sRNAs in the bacterial genome was performed using Infernal [24] software (http://eddylab.org/infernal/) and the Rfam database (https://rfam.xfam.org/).

Plasmid identification was performed using PlasmidFinder [25] (https://cge.cbs.dtu.dk/services/PlasmidFinder/) on the results of bacterial genome assembly by the plasmid sequences obtained, and then BLAST (https://blast.ncbi.nlm.nih.gov/Blast.cgi) software and PLSDB database (https://ccb-microbe.cs.uni-saarland.de/plsdb/) for plasmid annotation.

The Kyoto Encyclopedia of Genes and Genomes (KEGG, http://www.genome.jp/kegg) database was used for the annotation and classification of metabolic pathways [26], via the KEEG Automatic Annotation Server (KAAS, https://www.genome.jp/kegg/kaas/) [27].

### The average nucleotide identity (ANI) values and phylogenetic analyses

The average nucleotide identity (ANI) is based on the BLASTN algorithm. The phylogenetic tree was constructed using the TYGS server (https://tygs.dsmz.de) for taxonomic analyses based on complete genomes [28]. Specifically, the *P. pentosaceus* LL-07 genome was compared with conserved genomes of the same pedigree type, and the phylogenetic tree was visualized using iTol v.6.8.1.

### Comparative genomic analysis

All *P. pentosaceus* genomes were annotated with Prokaryotic Genome Annotation (Prokka) v.1.13 [29]. Subsequently, OrthoFinder v.2.5.4 [30] with default settings was used to identify orthogroups of orthologous gene pairs. Flip charts were generated using R v.4.3.2 and the UpsetR package [31]. The core orthogroups shared among *P. pentosaceus* strains were additionally annotated to Clusters Orthologous Groups (COG) categories [32] through the online server Batch CD-Search (https://www.ncbi.nlm.nih.gov/Structure/cdd/wrpsb.cgi) [33].

### Multiple genome alignments

The genomes of strains closely related to *P. pentosaceus* LL-07 were selected to enhance the analysis of the evolutionary relationships between species. The MCScanX [34] tool was used for overall protein-level alignment to examine the synteny among the genome sequences of each strain. Additionally, genome-wide visualization of coding sequence identity among 31 strains was performed using BLAST Ring Image Generator (BRIG) v.0.95 [35], with the *P. pentosaceus* ATCC25745 strain as the reference genome.

### Analysis of carbohydrates utilization and related genes

To examine the carbohydrate metabolism of *P. pentosaceus* LL-07, an experiment was conducted following the instructions provided by API50 CH (BioMerieux Diagnostics (Shanghai) Co., Ltd., China).

The genome of *P. pentosaceus* LL-07 was annotated using HMMER v.3.3.2, and the carbohydrate-active enzymes were analyzed by the Carbohydrate Active Enzymes Database (CAZy) (http://www.cazy.org/) [36, 37]. A cut-off limit of an *E*-value of 1e − 15 and a coverage of 0.35 were used to identify the CAZyme class.

### Prediction of the EPS Gene operon

The sequence of *P. pentosaceus* LL-07 was aligned with known EPS coding manipulators through the Basic Local Alignment Search Tool (BLAST) program [38–40] to identify the presence of the genes and map the EPS synthesis gene cluster.

### Mobile genetic elements

The PHASTER (PHAge Search Tool Enhanced Release) was used to identify and annotate prophages within all the strains [41]. The presence of clustered palindromic interspaced palindromic repeats (CRISPR) regions and CRISPR-associated (Cas) proteins were searched with CRISPRCasFinder (https://crisprcas.i2bc.paris-saclay.fr/) [42]. The TransposonPSI software was used to predicte the transposon [43].

### Safety assessment and identification of antibiotic resistance genes

Antimicrobial resistance genes (AMR) were studied using the Comprehensive Antibiotic Resistance Database (CARD) [44] and NCBI AMRFinder Plus [45] databases. Virulence factor genes were detected using the VFDB [46] database.

### Artificial saliva, gastric juice and intestinal juice tolerance

Overnight cultures of *P. pentosaceus* LL-07 were centrifuged and washed twice with physiological saline. The cells were resuspended in artificial saliva (pH 6.8) (Shanghai Yuanye Bio-Technology Co., Ltd, China) to tolerate 0.5 h (for a final cell concentration of approximately 10^8^ CFU/mL). The cells were collected by centrifugation and resuspended in artificial gastric juice (pH 3.0) (Shanghai Yuanye Bio-Technology Co., Ltd, China) for 2 h. Then, the cells were collected and resuscitated in artificial intestinal juice (pH 6.8) (Shanghai Yuanye Bio-Technology Co., Ltd, China) for 3 h. The culture conditions of each step were 37 ℃ with shaking (180 rpm) to simulate chewing and gastrointestinal conditions. The number of living cells was counted by the plate count method, and the survival rate of *P. pentosaceus* LL-07 was calculated.

### Bile salt tolerance

The collected cells of *P. pentosaceus* LL-07 were resuspended to a final cell concentration of approximately 10^8^ CFU/mL in physiological saline containing 0.2% bile salt (Sigma, America). After incubating for 3 h at 37 ℃, the bile tolerance was evaluated via the plate counting method.

### Autoaggregation assay [47]

The cells of *P. pentosaceus* LL-07 were centrifuged and washed twice with PBS, and resuspended to an optical density of 0.5 at 600 nm (A_0_). The suspensions were incubated at 37 ℃ for 4 h or 24 h without shaking. The aqueous phase was gently taken out to measure its absorbance at 600 nm (A_1_). Autoaggregation percentage was calculated as (1 − A_1_/A_0_) × 100.

### Hemolytic activity

The hemolytic activity of *P. pentosaceus* LL-07 was assessed with 5% (v/v) sheep red blood cells on trypsin agar (TSA). The strain was incubated at 37 ℃ for 24 h, followed by the measurement of α, β, or γ hemolysis [48]. Blank experimental and *S. aureus* ATCC6538 control groups were set up simultaneously.

### Antibiotic resistance

An antibiotic resistance test was performed on *P. pentosaceus* LL-07 following the instructions of the antibiotic sensitivity test paper method. The antibiotic sensitivity was assessed based on the size of the inhibition zone. The antibiotic sensitivity test paper and corresponding judgment criteria are detailed in Table 2.

**Table 2:** Determination standard of drug resistance test

Antibiotic Name	Content (μg/table)	Antibacterial ring diameter(mm)		
		R	I	S
Ampicillin	10	20	——	≥ 17
Penicillin	10	≤ 19	20–27	≥ 28
Chloramphenicol	30	≤ 12	13–17	≥ 18
Ofloxacin	5	≤ 12	13–15	≥ 16
Tetracycline	30	≤ 14	15–18	≥ 19
Doxycycline	30	≤ 12	13–15	≥ 16
Azithromycin	15	≤ 13	14–17	≥ 18
Erythromycin	15	≤ 13	14–22	≥ 23
Gentamicin	10	≤ 12	13–14	≥ 15
Kanamycin	30	≤ 12	13–14	≥ 15

Diameters of inhibition zones, mm (means ± SD of three trials) were interpreted as susceptible (S), moderately susceptible (I), and resistant (R)

### Antioxidant activity

The antioxidant activity was analyzed by measuring the DPPH free radical scavenging activity according to the methods of [49], with some modifications. The strains were cultured in MRS broth at 37 ℃ for 24 h and then centrifuged. The supernatant was mixed 1:1 (v/v) with ethanol DPPH solution (0.2 mmol/L) and incubated in the dark at 37 ℃ for 30 min. The absorbance (A) at 517 nm was measured (MultiskanGO, Thermo Fisher Scientific Inc., USA). The scavenging activity of DPPH was defined as scavenging activity (%) = [1 − (A_s_-A_b_)/A_c_] × 100%, where A_s_ is the A at 517 nm of the sample, A_b_ is the A of anhydrous ethanol instead of DPPH, and A_c_ is the A of water instead of sample.

### Statistical analysis

All the experiments were performed in triplicate, and the data are expressed as the means ± standard deviations that were analyzed using the Statistical Package for the Social Sciences (SPSS 19.0, IBM, Inc., Armonk, NY, USA).

The *P. pentosaceus* LL-07 genome sequence was submitted to the GenBank database, and the accession number PRJNA904022 was obtained.

## Results

### General characteristics of the *P. pentosaceus* LL-07 genome

**Fig.1:**
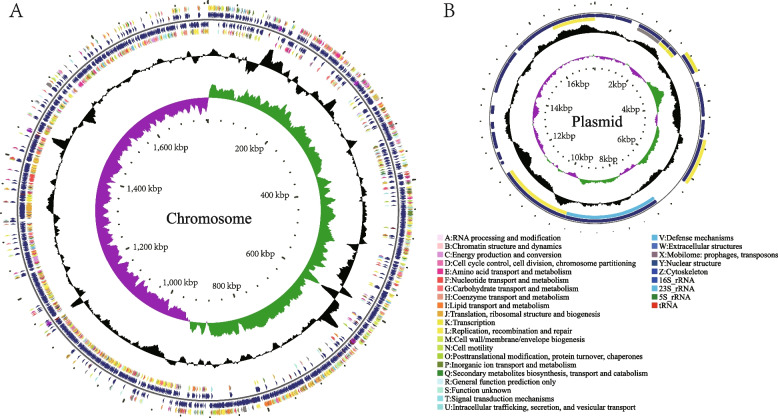
Chromosome (**A**) and plasmid (**B**) maps of *P. pentosaceus* LL-07. The circular illustration was visualized using the CGView server. In the cycle diagram, the first and fourth circles from the outside in represent CDS on the positive and negative strands, respectively, with different colors denoting different COG function classifications. The second and third circles represent CDS, tRNA, and rRNA on the positive and negative strands, respectively. The fifth circle indicates the GC content, and the sixth circle represents the GC-Skew value

The genome size of *P. pentosaceus* LL-07 is 1,782,685 bp, consisting of a 1,764,987 bp circular chromosome and a 17,698 bp circular plasmid. The GC content of the chromosome and plasmid are 37.18% and 33.08%, respectively (Table 3). The basic information and genomic features are shown in Fig. 1. There are 1,739 predicted chromosomal genes in *P. pentosaceus* LL-07, along with three kinds of non-coding RNAs: tRNA, rRNA, and sRNA (Supplementary Tables 1, 2 and 3).

**Table 3:** Genome characteristics of *P. pentosaceus* LL-07

Feature	Sequence Type	
	Chromosome	Plasmid
Sequence Topology	circular	circular
Sequence Number	1	1
Total Length(bp)	1,764,987	17,698
G + C content(%)	37.18	33.08
Gene number	1,714	25
tRNA Number	56	0
5S rRNA Number	5	0
16S rRNA Number	5	0
23S rRNA Number	5	0
sRNA Number	26	0

### Phylogenetic analysis

**Fig.2:**
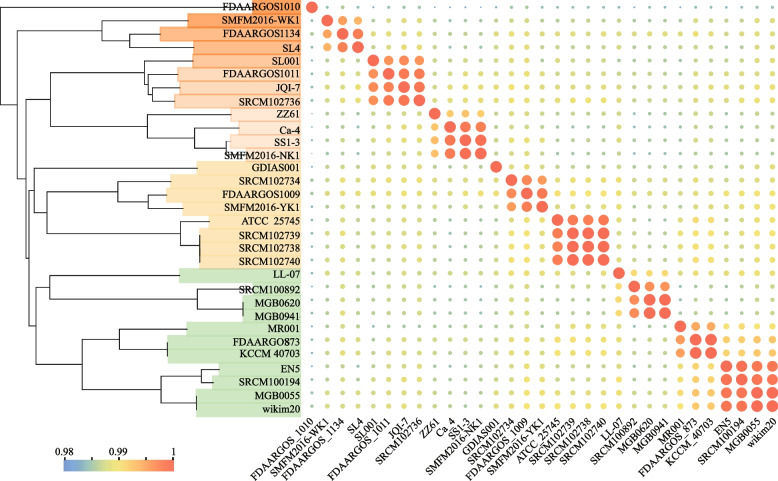
Phylogenetic analysis and ANI heatmap of *P. pentosaceus*. The proposed species cut-off boundary is above 98%, demonstrating identity within these strains

ANI analysis as a standard criterion for species classification and clustering. Previous research has shown that clear ANI divisions exist within and between species in most lineages. ANI values surpass 95% for the same species and fall below 95% for different species [50]. The results demonstrate that all 31 *P. pentosaceus* strains have ANI values exceeding 98%, with *P. pentosaceus* MGB0941 exhibiting the highest similarity to *P. pentosaceus* LL-07, with 99.02% ANI similarity. The phylogenetic tree (Fig. 2) also shows high similarity values among *P. pentosaceus* genomes. Additionally, ten strains were classified on the same branch as *P. pentosaceus* LL-07.

### Comparative genomic analysis

**Fig.3:**
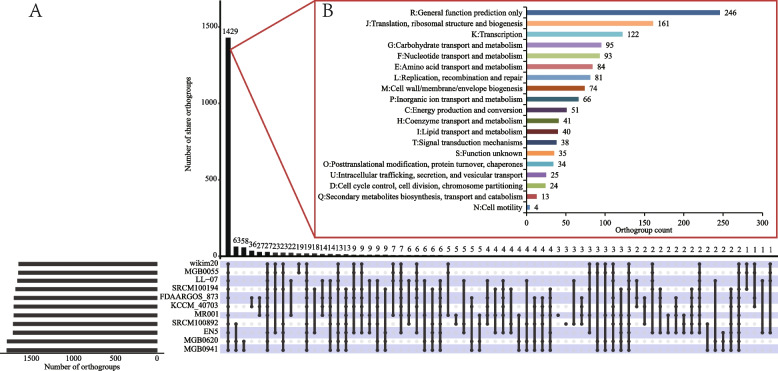
Upset plot (**A**) and COG classification (**B**) of orthogroups among the eleven *P. pentosaceus* species. The box plot illustrates the distributions of the mean orthogroup protein lengths. The bars represent the number of orthogroups exhibiting specific conservation patterns, with the numbers provided above each bar. The differently colored chart displays the functional categories of the core orthogroups based on COG classification using the Batch CD-Search server

Based on the phylogenetic tree results, ten bacterial strains were clustered with *P. pentosaceus* LL-07. A direct homologous gene analysis of these 11 strains included 19,324 genes, with 18,914 (97.88%) distributed into 2,053 orthogroups by OrthoFinder. Of these, 1,365 were identified as core orthogroups, and 688 were identified as accessory orthogroups (Fig. 3A). The orthogroups contained 1,675 (81.59%) genes of *P. pentosaceus* LL-07. The core orthogroups' additional details were classified into Clusters Orthologous Groups (COG) using the Batch CD-Search online server. Of the 1,365 core line types, 1,256 (92.01%) genes were categorized into the COG family (Fig. 3B), covering 21 functional classes.

### Multiple genome alignment

**Fig.4:**
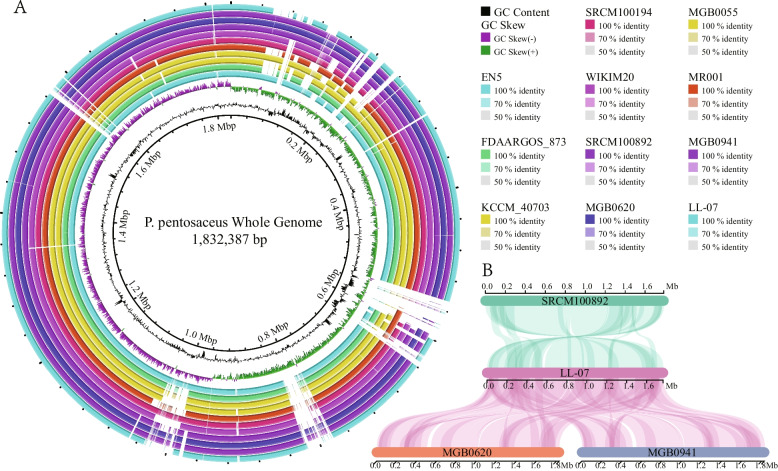
Multiple genome alignment of *P. pentosaceus* strains. **A** Nucleotide alignments of eleven *P. pentosaceus* genomes, generated with the BRIG. Different colors represent different strains. **B** Multiple genome alignment of *P. pentosaceus* LL-07, *P. pentosaceus* SRCM100892, *P. pentosaceus* MGB0620 and *P. pentosaceus* MGB0941. Visualization of alignment is organized into one horizontal panel per genome sequence, with the label of the genome sequence name at the center of each panel. Homologous blocks within the genome are connected by lines

For a comprehensive visualization of the coding sequences between *P. pentosaceus* LL-07 and the other 30 strains, the genomes of the 30 strains were compared to the reference genome *P. pentosaceus* ATCC25745.. The solid section of the circle represents similarities between the genomes, while the blank areas indicate variability (Fig. 4A). The comparison result shows the presence of the open reading frame in the reference genome, which was absent in the remaining genomes, and could be attributed to differences in assembly quality [51].

*P. pentosaceus* SRCM100892, *P. pentosaceus* MGB0620, and *P. pentosaceus* MGB0941 were selected for multi-genome alignment (Fig. 4B) due to their close relationships with *P. pentosaceus* LL-07 in the phylogenetic tree (Fig. 2). There was a relatively high sequence similarity between *P. pentosaceus* LL-07 and *P. pentosaceus* MGB0620, as well as *P. pentosaceus* MGB0941. The positions of similarity between *P. pentosaceus* LL-07 and *P. pentosaceus* SRCM100892 mainly involve inversions within the chromosome. Moreover, there are regions in *P. pentosaceus* LL-07 that do not align with *P. pentosaceus* SRCM100892, *P. pentosaceus* MGB0620, and *P. pentosaceus* MGB0941. This illustrates the divergence of one strain of the same species from others and provides substantial evidence for the reconstruction of *P. pentosaceus* LL-07.

### Genotype analysis for carbohydrate utilization in *P. pentosaceus* LL-07

**Fig.5:**
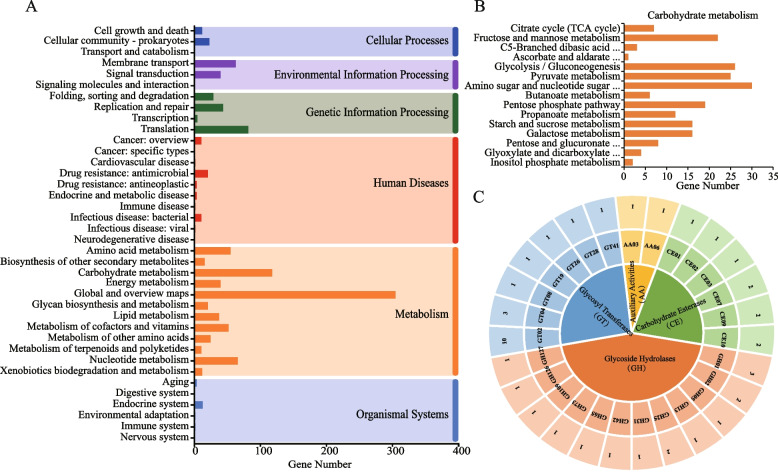
Genotype analysis for carbohydrate utilization in *P. pentosaceus* LL-07. **A** Kyoto Encyclopedia of Genes and Genomes (KEGG) functional annotation. The functional categories are represented as follows: Dark Blue-Cellular Processes; Purple-Environmental Information Processing; Green-Genetic Information Processing; Red-Human Diseases; Orange-Metabolism;Blue-Organismal Systems. **B** Distribution of CAZymes. Different colors represent different classes of CAZymes identified in the genome. The representation from the inner to outer rings are, CAZyme classes, CAZyme families, and the number of genes identified in each family, respectively. **C** Carbohydrate metabolism

In the whole genome sequence of *P. pentosaceus* LL-07, 956 genes were functionally annotated by the KEGG database (54.97%), participating in six major classes of metabolic pathways (Fig. 5A). Among the metabolism-related genes, the most abundant were those associated with Global and Overview Maps (305 genes), followed by Carbohydrate Metabolism (118 genes), Nucleotide Metabolism (66 genes) and Amino Acid Metabolism (55 genes).

Carbohydrate active enzymes (CAZymes) are classified based on the sequences of enzymes involved in complex carbohydrate metabolism. These enzymes were studied by comparing the predicted genes in the *P. pentosaceus* LL-07 genome with the CAZY database. There were forty-five genes were identified across four classes of CAZymes (Fig. 5B)..

*P. pentosaceus* LL-07 can utilize 25 out of 49 carbohydrates more effectively (Table 4). In addition to monosaccharides and disaccharides, which are typically degraded by *Pediococcus*, *P. pentosaceus* LL-07 has the ability to utilize lactose and galactose but not polysaccharides such as starch or glycogen. Genes related to carbohydrate metabolism were identified in the *P. pentosaceus* LL-07 metabolic pathway, as shown in Fig. 5C and Supplementary Table 4. The greatest number of genes (30) were involved in Amino Sugar and Nucleotide Sugar Metabolism, followed by genes in Glycolysis/Gluconeogenesis(26 genes), Pyruvate Metabolism (25 genes), Fructose and Mannose Metabolism (22 genes), Pentose Phosphate Pathway (22 genes), and Pentose-phosphate Metabolism (25 genes), Phosphate Pathway (19), Starch and Sucrose Metabolism (16), Galactose Metabolism (16), and Propanoate Metabolism (12).

**Table 4:** Sugar fermentation of *P. pentosaceus* LL-07

No	Substrate name	Result	No	Substrate name	Result	No	Substrate name	Result
0	Control	-	17	INOsitol	-	34	D-MeLeZitose	+
1	GLYcerol	-	18	MANnitol	+	35	D-RAFfinose	+
2	ERYthritol	-	19	SORbitol	+	36	Starch	-
3	D-ARAbinose	-	20	Methyl-*α*D-Mannopyranoside	+	37	GLycoGen	-
4	L-ARAbinose	+	21	Methyl-*α*D-Glucopyranoside	+	38	XyLiTol	-
5	D-RIBose	+	22	N-Acetyl Glucosamine	+	39	D-GENtiobiose	+
6	D-XYLose	+	23	AMYgdalin	+	40	D-TURanose	+
7	L-XYLose	-	24	ARButin	+	41	D-LYXose	-
8	D-ADOnitol	-	25	Esculin ferric citrate	-	42	D-TAGatose	+
9	Methyl-*β*D-Xylopyranoside	-	26	SALicin	+	43	D-FUCose	-
10	D-GALactose	+	27	D-CELlobiose	+	44	L-FUCose	-
11	D-GLUcose	+	28	D-MALtose	+	45	D-ARabitoL	-
12	D-FRUctose	+	29	D-LACtose	+	46	L-ARabitoL	-
13	D-MaNnosE	+	30	D-MELibiose	+	47	GlucoNaTe	?
14	L-SorBosE	-	31	D-Sucrose	+	48	2 Keto Gluconate	?
15	L-RHAmnose	-	32	D-TREhalose	+	49	5 Keto Gluconate	-
16	DULcitol	-	33	INUlin	-			

“ + ” was interpreted as a positive reaction, “-” was interpreted as a negative reaction, and “?” was interpreted as a reaction that could not be determined

### Prediction of the EPS gene operon in *P. pentosaceus* LL-07

**Fig.6:**
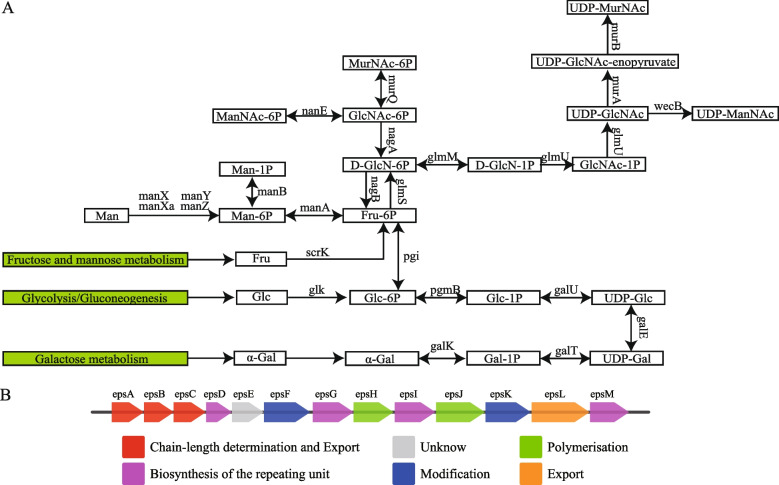
Prediction of the EPS Gene Operon in *P. pentosaceus* LL-07. **A** Amino sugar and nucleotide sugar metabolism in *P. pentosaceus* LL-07. The red box indicates potential exopolysaccharide synthesis monomers. **B** EPS synthesis pathway. Different colors represent different functions of genes

Amino and nucleotide sugars serve as precursors for EPS synthesis. In *P. pentosaceus* LL-07, aminosugars and nucleotide sugars are predominantly represented by uridine diphosphate sugars (UDP sugar). The synthesis pathways and related genes are depicted in Fig. 6A.

*P. pentosaceus* LL-07 contains a gene cluster consisting of 13 genes (12.77 kb) responsible for EPS synthesis (Fig. 6B). These genes included chain-length determination and export genes (*gene0578, gene0579* and *gene0580*), genes involved in the biosynthesis of the repeating unit (*gene0581, gene0584, gene0586* and *gene0590*), modification genes (*gene0583* and *gene0588*), polymerization genes (*gene0585* and *gene0587*), and export genes (*gene0589*).

### Mobile genetic elements in *P. pentosaceus* LL-07

Prephages, commonly found in bacterial genomes, represent mild phage genomes integrated into the bacterial genome [52]. They often contain functional genes, such as antibiotic resistance and virulence genes, significantly to enhance bacterial competitiveness and the ability to withstand adverse environments [53]. Two potential prophages were identified in the *P. pentosaceus* LL-07 genome (Supplementary Table 5), with phage 2 being the largest at 29.62 Kb, encoding 37 genes. Phage2 includes genes related to phage formation and those associated with lysozyme M1, acetyl coenzyme A carboxylase, and amino acid permease. Notably, no pathogenesis-related genes were detected in either prophage 1 or prophage 2.

The CRISPR/Cas system functions as a defense mechanism in bacteria against foreign genetic material, such as phage viruses and foreign plasmids. [54, 55]. It recognizes exogenous DNA and silences the expression of exogenous genes. CRISPR usually consists of numerous short and conserved repeating sequence regions (repeat) and spacers. Cas, a double-stranded DNA nuclease, resides near CRISPR sites and cleaves the target site under the guidance of guide RNA. The CRISPR-Cas system is formed by clusters of CRISPRs along with Cas proteins. In the *P. pentosaceus* LL-07 genome, two dispersed CRISPRs and three dispersed Cas were identified, as detailed in Supplementary Table 6.

Transposons are DNA sequences in the genome that can move between chromosomes, plasmids, or phages in bacteria. The genome of *P. pentosaceus* LL-07 was analyzed, revealing the presence of three transposons (Supplementary Table 7). These transposons belong to the LINE family (Tn01, Tn03) and the helitronORF family (Tn02). Tn01, Tn02, and Tn03 encode a ribonuclease H family protein, a primosomal protein N', and an NAD-dependent DNA ligase LigA, respectively. These genes are involved in DNA replication and transcription.

### Safety assessment and identification of antibiotic resistance genes

There were 163 virulence factor-related genes in *P. pentosaceus* LL-07 (Supplementary Table 8). In comparison, the predicted virulence factors are primarily associated with antiphagocytosis (28), the iron uptake system (24), adherence (17), regulation (13), and toxins (13). Notably, the 13 toxin-related virulence genes predominantly function in self-protection and resistance, with no identified gene capable of toxin production. Prediction of resistance genes identified eighty-six genes associated with resistance to twenty-five antibiotics (Supplementary Table 9).

### Assessment of probiotic properties of *P. pentosaceus* LL-07

The probiotics of *P. pentosaceus* LL-07 were evaluated, including tolerance of the simulated digestion process, autoaggregation assay, hemolytic activity, antibiotic resistance, and antioxidant activity. It was observed that *P. pentosaceus* LL-07 maintained better viability than *P. pentosaceus* ATCC25745 in artificial saliva (*p* < 0.05). Both *P. pentosaceus* LL-07 and *P. pentosaceus* ATCC25745 showed tolerance to artificial gastric juice, intestinal juice, and bile salt (Table 5). At 4 h, *P. pentosaceus* LL-07 exhibited higher autoaggregation than *P. pentosaceus* ATCC25745 (*p* < 0.05) (Table 5). There was no significant difference in autoaggregation between the two strains after 24 h (*p* > 0.05). The results indicate that both strains possess DPPH radical scavenging ability, with *P. pentosaceus* LL-07 exhibiting a higher ability than *P. pentosaceus* ATCC25745 (*p* > 0.05). This difference in scavenging ability may be attributed to the differences in the structure and composition of metabolites secreted by the two strains.

**Table 5:** Probiotic properties of *P. pentosaceus* LL-07

Probiotic Properties	LL-07	ATCC 25745
Survival in Oral and GI tract, %		
Artificial Saliva		
0 h	100.00	100.00
0.5 h	103.33 ± 2.63*	99.07 ± 1.31
Artificial Gastric Juice		
0 h	100.00	100.00
2 h	89.16 ± 0.49	88.84 ± 0.17
Artificial Intestinal Juice		
0 h	100.00	100.00
3 h	77.64 ± 1.74	78.02 ± 0.88
Bile salt		
0 min	100	100.00
3 h	66.45 ± 1.13*	59.24 ± 2.31
Auto-aggregation, %		
4h	62.4 ± 1.12*	43.78 ± 0.91
24h	73.73 ± 1.38	74.05 ± 0.88
Antioxidant activity, %	94.12 ± 3.21*	73 ± 4.14

^*^means statistically significant difference (*p* < 0.05) as compared to the reference strain *P. pentosaceus* A

**Fig.7:**
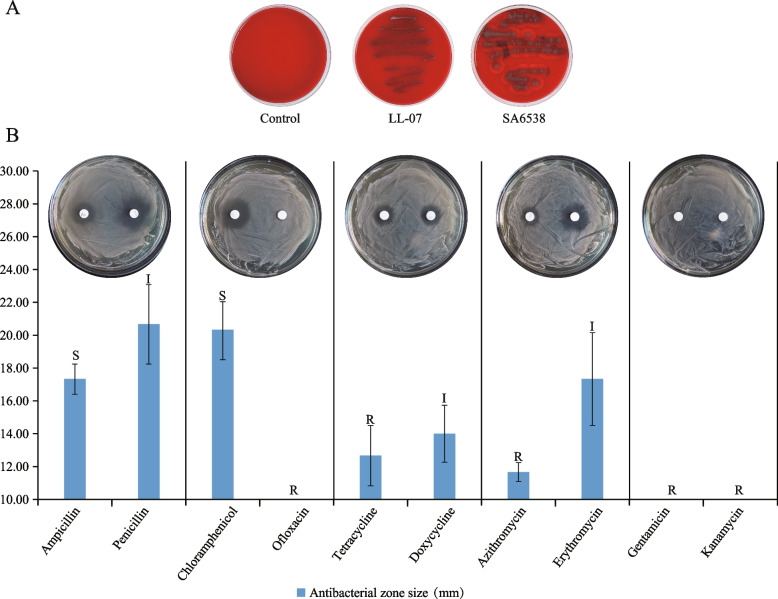
Safety assessment of *P. pentosaceus* LL-07. **A** Hemolysis. **B** Antibiotic resistance.The above picture shows the results of antibiotic resistance experiment in each group. The bottom square column shows the diameter of antibacterial zone for each group. The letters above the bar indicate resistance to antibiotics. Where R stands for resistant, I for moderately susceptible, and S for susceptible

The hemolysis experiment demonstrates that *P. pentosaceus* LL-07 forms milky-white colonies without a hemolytic circle after 48 h of incubation on blood plates, indicating γ-hemolysis without virulence (Fig. 7A). Antibiotic resistance experiments revealed differences in *P. pentosaceus* LL-07 resistance to different antibiotics. Notably, within the same antibiotic class, diverse resistance patterns were observed (Fig. 7B).

## Discussion

This study involved subjecting *P. pentosaceus* LL-07 to whole genome sequencing, genome characterization, and comparative analysis. The mean G + C content of *P. pentosaceus* LL-07 is 37.18%, which is a standard feature of bacterial taxonomy and reflects evolutionary relatedness. This value aligns with Bergey’s Manual of Systematics of Archaea and Bacteria [56]. The bacterial genome size was 1.78 Mb with 1,739 CDS. Additionally, the bacterium contained a circular plasmid of 17 kb..

ANI has been served as the gold standard for genetic restriction in prokaryotic species [57]. In the ANI analysis, *P. pentosaceus* LL-07 was compared to 30 strains of *P. pentosaceus*, and classified to the same branch with ten strains. Notably, the ANI values of *P. pentosaceus* MGB0620, *P. pentosaceus* MGB0941, and *P. pentosaceus* SRCM100892 were relatively narrow with *P. pentosaceus* LL-07, indicating limited genetic diversity. The results suggest that *P. pentosaceus* LL-07 may not require an extensive set of genes to adapt to diverse ecological niches. Phylogenetic similarities between the *P. pentosaceus* LL-07 genome and 30 *P. pentosaceus* strains from NCBI were revealed through ANI and genome-wide analyses. The genome of *P. pentosaceus* contains 1,365 directly homologous genes, which are primarily involved in maintenance functions crucial for the growth and survival of the species. Multiple genome analyses revealed that strains resembling those in the phylogenetic tree exhibited similar levels of gene deletion..

The carbohydrate utilization capacity of bacteria is a crucial indicator of strain functionality, forming the basis for strain culture and selection. *P. pentosaceus* LL-07 exhibits superior carbohydrate utilization, possibly due to the presence of numerous genes encoding potential sugar transporters. Typically, the genus *P. pentosaceus* cannot ferment lactose [58, 59]. Sugar fermentation experiments and KEGG pathway analysis revealed a complete lactose metabolic pathway in *P. pentosaceus* LL-07, which can better utilize lactose and galactose. Upon entering the bacterium, lactose can be broken down into galactose (D-Gal) and glucose by the β-galactosidase LacZ (*gene0193*, *gene0194*). Under the catalysis of galactose changing enzymes GalM (gene0191, gene0210, gene1010), galactose kinases GalK (gene0116, gene0195), and glucose hexose-1-phosphate uridine transferase GalT (gene0197), galactose forms uridine diphosphate galactose (UDP Gal). UDP-Gal can be converted into two different compounds: uridine diphosphate-furanogalactose (UDP-Galf) and uridine glucose diphosphate (UDP-Glc). UDP-Galf is produced by the uridine diphosphate-furanogalactose metathesis enzyme Glf (gene0588) and is used for the biosynthesis of N-glycans. UDP-Glc, on the other hand, is formed by the catalytic action of uridine diphosphate-glucose 4-differential isomerase GalE (gene0196, gene1424) and enters a subsequent metabolic pathway. *P. pentosaceus* LL-07 is a natural strain capable of fermenting lactose, which contradicts the previous belief that lactose-positive strains do not exist in naturally occurring *Pediococcus*. This conclusion is supported by the study of Jiang [51] and overturns the notion that lactose-positive strains are absent in natural *Pediococcus* [59].

CAZyme analysis revealed that *P. pentosaceus* LL-07 predominantly contains GTs, GHs, CEs, and AAs, which are crucial for glucose metabolism. GTs are enzymes that have substrate specificity and catalyze the transfer of sugar groups from activated nucleotide sugar donors to lipids, polysaccharides, or protein acceptors. In *P. pentosaceus* LL-07, GTs are classified into seven different families. Enzymes from GT2 and GT4 families make up 72.22% of the total GTs. These enzymes convert glucose, mannose, xylose, galactose, trehalose, arabinose, etc., into metabolites for metabolic utilization. GHs represent key enzymes for carbohydrate metabolism and play a pivotal role in the hydrolysis of carbohydrate glycosidic bonds. *P. pentosaceus* LL-07 contains a total of 12 different GH families that act on glycosidic bonds formed by glucose, mannose, xylose, galactose, trehalose, arabinose, etc. This diversity proves advantageous for the strain's growth in various environments, making it versatile and critical for development [60, 61]. GTs and GHs are closely related to EPS precursor synthesis. The analysis showed that *P. pentosaceus* LL-07 has numerous GHs and GTs genes associated with mannose metabolism, indicating that mannose may be a significant component of the synthesized EPS. Additionally, the GHs include two lysozymes (GH25 or GH73), which enhance the bacteria's ability to adapt and resist external challenges [62, 63]. These findings align with the study conducted by Oliveira et al. [64].

In addition to bacteriocins and bacteriocin-like inhibitory substances (BLIS), EPS is an important metabolite secreted by *P. pentosaceus* with specific functions. *P. pentosaceus* DPS has been reported to synthesize heat-resistant EPS in the human gastrointestinal tract. This EPS destructs biofilms originating from harmful pathogens by inhibiting adhesion, thereby combating *Listeria monocytogenes* and *Escherichia coli* [65]. *P. pentosaceus* M41, sourced from marine environments, secretes EPS-M41 exhibiting antitumor activity, demonstrating high efficacy against Caco-2 and MCF-7 cells [66]. Moreover, certain strains of *P. pentosaceus*, such as *P. pentosaceus* Be1 (isolated from fermented foods) [67] and *P. pentosaceus* S-SU6 (isolated from the intestines of blue mackerel) [68], specialize in scavenging hydrogen peroxide. Due to the secretion of acidic EPS, *P. pentosaceus* MYU 759 demonstrates hydroxyl radical antioxidant capacity (HORAC), proving beneficial for its isolation from soy milk yogurt as an antioxidant product [69].

The prediction of the EPS synthesis gene cluster in *P. pentosaceus* LL-07 using antiSMASH did not yield a matching gene cluster model. However, upon relaxing the detection requirements, genes related to EPS synthesis were identified between chromosomes 596,709 and 609,483. Moreover, by utilizing Orthomcl software and BlastN to predict the gene operons of EPS, it was determined that the 13 ORFs in the EPS cluster of *P. pentosaceus* LL-07 are related to the EPS biosynthesis process. It can be inferred that the genes on the *P. pentosaceus* LL-07 EPS synthesis gene cluster exhibit specific structural differences from known gene clusters but encompass essential elements of EPS synthesis, including regulatory genes, chain length-determining genes, repeat unit biosynthesis genes, and aggregation output genes.

The predicted products of *gene0578*, *gene0579*, and *gene0580* are homologous to Wzz, the CpsD/CapB family tyrosine protein kinase, and the CpsB/CapC family capsule biosynthesis tyrosine phosphatase, respectively. The literature reports indicate that Wzz is one of the factors influencing the heterogeneity of the *E. coli* O antigen chain length [70]. The CpsD/CapB family tyrosine protein kinase is a critical regulatory device in bacterial physiology and acts as a domain protein involved in EPS biosynthesis [71]. The CpsB/CapC family of capsule biosynthetic tyrosine phosphatases belongs to the protein tyrosine phosphatase (PTP) superfamily and can synergistically interact with protein tyrosine kinases. In *E. coli* K-30,the tyrosine kinase Wzc and its homologous phosphatase Wzb are critical factors in the synthesis and assembly of capsule polysaccharides. In the gram-positive bacterium *Streptococcus pneumoniae*, the CpsCD complex is similar to Wzc, while the phosphatase CpsB is the corresponding homologous phosphatase [72]. In *S. pneumoniae*, CpsB regulates the biosynthesis of capsule polysaccharides through tyrosine dephosphorylation of its homologous tyrosine kinase CpsD [73]. The predicted products of *gene0581* and *gene0586* are homologous to sugar transfer. These enzymes can catalyze the transfer of sugars from donors (such as UDP-glucose or UDP-galactose) to lipid carriers (such as undecyl phosphate) [74] and sugar transferases similar to the *E. coli* beta-1,6-galactofuranosyltransferase WbbI, which is involved in transferring galactofuranose (Galf) onto an alpha-D-gluco-configured acceptor substrate to form a beta-1,6-linkage [75]. The biosynthesis of EPS repeat units also requires multiple glycosyltransferases (GTs), whose coding genes typically reside in the middle of EPS gene clusters [76]. *Gene0584* and *gene0590* were identified as GT genes. Although predicted functions of the GT genes in *P. pentosaceus* LL-07 are the same (glycosyltransferase), their nucleotide sequences exhibit some variation. Therefore, the activity of these glycosyltransferases may vary [77], offering potential avenues for enhancing EPS production through genetic modification. *Gene0585* has been identified as a Stealth CR1 domain-containing protein, and research indicates that this protein may be a D-hexose-1-physiological transfer involved in EPS biosynthesis [78]. The predicted product of the precursor gene (*gene0588*) is associated with UDP-galactopyranose mutase. This enzyme is involved in converting UDP-GALP into UDP-GALF through a 2-keto intermediate. UDP-galactopyranose mutase regulates the biosynthesis of precursors in lipopolysaccharide production in *Klebsiella pneumoniae* [79]. *Gene0587* and *gene 0589* are associated with polysaccharide polymerase and polysaccharide biosynthesis C-terminal domain-containing proteins associated with polysaccharide polymerization and export [80, 81].

Mobile genetic elements, such as prephages and transposons, are frequently present in microorganisms. They can serve as carriers of pathogenic genes or facilitate horizontal gene transfer (HGT) from antimicrobial-resistant (AMR) microorganisms to others [82]. Therefore, it is necessary to conduct further analysis of these regions to identify any undiscovered pathogenic or AMR genes in the genome of *P. pentosaceus* LL-07. The genome contains two potential prophages and three transposons. However, no pathogenicity-related genes were detected. Phage 2 includes a complete prophage region and genes related to lysozyme M1, acetyl-CoA carboxylase, and amino acid permease. The transposon genes are mainly related to DNA replication and transcription.

The CRISPR-Cas system serves as an adaptive immune role against invasive elements, such as phages in bacterial cells, and regulates gene activity, DNA repair, and genome recombination [55, 83]. Despite the presence of two isolated CRISPRs and three isolated clusters of Cas3 in its genome, the predicted results suggest that the CRISPR-Cas system may not be present in the genome of *P. pentosaceus* LL-07. According to Zhang and Ye [84], CRISPR regions without cas genes may not be functional or work with distant cas motifs in the same genome.

During proliferation, certain bacteria can produce hemolysin, which specifically binds to red blood cells, causing severe hemolytic reactions and resulting in conditions such as anemia. Hemolysis test results indicated that in comparison to the *S. aureus* ATCC6538 control group, *P. pentosaceus* LL-07 exhibited general growth on the blood plate, without the formation of a hemolytic circle, indicating nontoxic γ-hemolysis. Accordingly, in *P. pentosaceus* LL-07, potential virulence factor genes were identified using the Virulence Factor Database (VFDB), revealing the presence of 163 putative virulence factor genes. Most of these genes exhibited less than 70% similarity in the VFDB, indicating low resemblance to genes in the database. Additionally, a search and Blast comparison revealed the absence of genes encoding toxins, such as hemolysin, in *P. pentosaceus* LL-07, consistent with the experimental findings.

AMR analysis revealed 86 antibiotic resistance (AR) genes in the *P. pentosaceus* LL-07 genome, corresponding to 25 antibiotic. The results of the antibiotic resistance experiment were generally consistent with the genetic analysis. Aminoglycoside and fluoroquinolone antibiotic genes were present in the *P. pentosaceus* LL-07 genome, resulting in resistance to gentamicin, kanamycin, and ofloxacin. Notably, despite the presence of macrolide and tetracycline antibiotic genes in the *P. pentosaceus* LL-07 genome, the strain exhibited moderate sensitivity to erythromycin and doxycycline, and no sensitivity to azithromycin and tetracycline. No genes related to penicillin antibiotics were identified, indicating sensitivity and moderate sensitivity to ampicillin and penicillin, respectively. This variation could be due to multiple factors, such as gene expression levels and substrate specificity of the expression products [85].

Further analysis of the *P. pentosaceus* LL-07 genome revealed that theresistance mechanism of this strain predominantly involves antibiotic efflux, antibiotic target alteration, and antibiotic target protection. These systems may contribute to bacterial resistance to these antibiotics. The main concern about genes that confer AR in LAB is their potential to be transferred to other bacteria, mainly pathogens, which could lead to complications and reduce the effectiveness of antibiotic treatment. No AR genes were identified by analyzing the prophage region, transposons and plasmid genes in the *P. pentosaceus* LL-07 genome, indicating a low risk of transfer to other bacteria.

*P. Pentosaceus* LL-07 exhibits bile salt and digestive juice tolerance, adhesion, and antioxidation. These characteristics are supported by 1 Choloylglycine hydrolase family protein gene, eight F0F1 ATP synthase genes, nine adhesion-related genes, and 31 antioxidant-related genes (Supplementary Table 10). Additionally, *P. pentosaceus* LL-07 contains a gene that encodes bifunctional folate polyglutamate synthase/dihydrofolate synthetase, which is associated with folate synthesis. The gene can code bifunctional folate polyglutamate synthase/dihydrofolate synthetase, which can catalyze the synthesis of dihydrofolate and folylpolyglutamate. Dihydrofolate is a precursor to folate, and folylpolyglutamate is the primary form of folate in the body.

## Conclusion

In this study, the probiotic potential of the meat-derived *P. pentosaceus* LL-07 was studied via whole genome analysis combined with experimental studies. In this study, we determined the accurate taxonomic location, carbohydrate utilization capacity, safety and probiotic characteristics of *P. pentosaceus* LL-07. The whole genome sequence of *P. pentosaceus* LL-07 provides potential for new research to determine the genetic factors and mechanisms related to its beneficial role as a probiotic. The in vitro experimental results of the probiotic characteristics revealed its high tolerance to saliva, gastric juice, intestinal juice and bile, auto-aggregation, safety, antibiotic resistance and antioxidant activity. Compared with the reference strain, *P. pentosaceus* LL-07 has better saliva and bile tolerance, faster autoaggregation and greater antioxidant activity. In conclusion, genomic analysis and in vitro experiments revealed that *P. pentosaceus* LL-07 is a strong candidate for probiotics. Future research will focus on the relationship between EPS and probiotic activity to provide a deeper understanding of its probiotic mechanism, promoting the future development of probiotic products.

### Supplementary Information

**Supplementary Material 1. **

## Data Availability

The genome sequence was submitted to the GenBank database, and the accession number PRJNA904022 was obtained. All other data generated are included in the supplemental material files.
