# Supplementary material for: Genomic characterization and probiotic potential assessment of an exopolysaccharide-producing strain Pediococcus pentosaceus LL-07 isolated from fermented meat
Source: BMC Microbiol. 2024 Apr 25;24:142. doi: 10.1186/s12866-024-03304-6 (PMC11044368; doi:10.1186/s12866-024-03304-6)
Supplement: Supplementary file 1 — Supplementary Material 1. [file 12866_2024_3304_MOESM1_ESM.pdf]

Supplementary Materials

Genomic characterization and probiotic potential assessment of an exopolysaccharide-producing strain *Pediococcus pentosaceus* LL-07 isolated from fermented meat

Kuan Lu[a,b], Xueya Wang[c], Ying Zhou[b], Qiujin Zhu[a,b*]

[a] Key laboratory of Plant Resource Conservation and Germplasm Innovation in Mountainous Region (Ministry of Education), College of Life Sciences/Institute of Agro-bioengineering, Guizhou University, Guiyang 550025, Guizhou Province, China.
[b] Guizhou Province Key Laboratory of Agricultural and Animal Products Storage and Processing, School of Liquor and Food Engineering, Guizhou University, Guizhou, Guiyang, 550025, China
[c] Chili Pepper Research Institute, Guizhou Provincial Academy of Agricultural Sciences, Guizhou, Guiyang, 550006, China

* Corresponding author

E-mail address:ls.qjzhu@gzu.edu.cn

CONTEST

Supplementary Table 1 Prediction of tRNA in *P. pentosaceus* LL-07 2
Supplementary Table 2 Prediction of rRNA in *P. pentosaceus* LL-07 4
Supplementary Table 3 Prediction of sRNA in *P. pentosaceus* LL-07 5
Supplementary Table 4 Carbohydrate metabolism in *P. pentosaceus* LL-07 6
Supplementary Table 5 Gene details of prophage in *P. pentosaceus* LL-07 9
Supplementary Table 6 Prediction of CRISPR/Cas in *P. pentosaceus* LL-07 11
Supplementary Table 7 Prediction of Transposon in *P. pentosaceus* LL-07 12
Supplementary Table 8 Detailed table of virulence gene prediction in *P. pentosaceus* LL-07 13
Supplementary Table 9 Statistical table of prediction and classification of antibiotic resistance genes in *P. pentosaceus* LL-07 17
Supplementary Table 10 The probiotic-related genes in *P. pentosaceus* LL-07 18

Supplementary Table 1 Prediction of tRNA in *P. pentosaceus* LL-07

tRNA ID	Startbp	Endbp	tRNA Type	Anti Codon	Score
LL-07.tRNA01	244262	244335	Thr	GGT	75.24
LL-07.tRNA02	645925	645999	Ile	GAT	91.61
LL-07.tRNA03	646005	646077	Ala	TGC	84.82
LL-07.tRNA04	649404	649476	Val	TAC	88.44
LL-07.tRNA05	649486	649558	Lys	TTT	85.34
LL-07.tRNA06	649618	649690	Thr	TGT	86.9
LL-07.tRNA07	649701	649772	Gly	GCC	79.21
LL-07.tRNA08	649805	649890	Leu	TAA	65.63
LL-07.tRNA09	649899	649972	Arg	ACG	67.59
LL-07.tRNA10	649982	650055	Pro	TGG	88.17
LL-07.tRNA11	650095	650168	Met	CAT	77.61
LL-07.tRNA12	650189	650262	Met	CAT	90.44
LL-07.tRNA13	650309	650398	Ser	TGA	63.3
LL-07.tRNA14	650408	650481	Met	CAT	77.81
LL-07.tRNA15	650486	650559	Asp	GTC	76.71
LL-07.tRNA16	650565	650637	Phe	GAA	73.75
LL-07.tRNA17	650662	650732	Gly	TCC	71.75
LL-07.tRNA18	650751	650825	Ile	GAT	91.61
LL-07.tRNA19	650829	650916	Ser	GCT	63.03
LL-07.tRNA20	650940	651011	Glu	TTC	66.65
LL-07.tRNA21	651045	651118	Met	CAT	77.81
LL-07.tRNA22	651123	651196	Asp	GTC	76.71
LL-07.tRNA23	667965	668048	Leu	CAG	60.3
LL-07.tRNA24	677769	677839	Gly	CCC	65.08
LL-07.tRNA25	817680	817753	Asn	GTT	80.23
LL-07.tRNA26	1065580	1065667	Ser	CGA	70.45
LL-07.tRNA27	1268928	1269000	Glu	CTC	59.24
LL-07.tRNA28	1269009	1269081	Gln	CTG	68
LL-07.tRNA29	1708262	1708334	Lys	CTT	84.99
LL-07.tRNA30	1443949	1443875	Ile	GAT	91.61
LL-07.tRNA31	1443869	1443797	Ala	TGC	84.82
LL-07.tRNA32	1440469	1440397	Asn	GTT	79.59
LL-07.tRNA33	1440348	1440276	Thr	CGT	74.08
LL-07.tRNA34	1437207	1437122	Tyr	GTA	75.5
LL-07.tRNA35	1437118	1437047	Gln	TTG	64.93
LL-07.tRNA36	1424809	1424724	Leu	AAG	58.01
LL-07.tRNA37	1348046	1347974	Val	TAC	87.25
LL-07.tRNA38	1347931	1347850	Leu	TAG	63.26
LL-07.tRNA39	1347837	1347765	Thr	TGT	86.05
LL-07.tRNA40	1347760	1347689	Gly	GCC	79.21
LL-07.tRNA41	1347667	1347594	Arg	ACG	69.22
LL-07.tRNA42	1347503	1347430	Pro	CGG	70.99
LL-07.tRNA43	1148771	1148699	Asn	GTT	79.59
LL-07.tRNA44	1148686	1148595	Ser	GGA	58.11
LL-07.tRNA45	1148576	1148505	Glu	TTC	66.65
LL-07.tRNA46	1148497	1148425	Val	TAC	87.25
LL-07.tRNA47	1148406	1148333	Asp	GTC	76.71
LL-07.tRNA48	1148310	1148238	Phe	GAA	73.75
LL-07.tRNA49	1148193	1148120	Trp	CCA	64.7
LL-07.tRNA50	1148114	1148042	His	GTG	64.51

LL-07.tRNA51	1147994	1147911	Leu	CAA	62.57
LL-07.tRNA52	1147826	1147756	Cys	GCA	71.31
LL-07.tRNA53	753385	753312	Arg	TCT	81.51
LL-07.tRNA54	621983	621910	Arg	CCT	75.06
LL-07.tRNA55	468377	468306	Arg	CCG	49.87
LL-07.tRNA56	94149	94077	Ala	CGC	79.98

Supplementary Table 2 Prediction of rRNA in *P. pentosaceus* LL-07

rRNA ID	Location	Startbp	Endbp	Strand	rRNA Type
LL-07.rRNA01	Chromosome	103431	105002	+	16S_rRNA
LL-07.rRNA02	Chromosome	105234	108153	+	23S_rRNA
LL-07.rRNA03	Chromosome	108253	108364	+	5S_rRNA
LL-07.rRNA04	Chromosome	239210	240781	+	16S_rRNA
LL-07.rRNA05	Chromosome	241013	243932	+	23S_rRNA
LL-07.rRNA06	Chromosome	244032	244143	+	5S_rRNA
LL-07.rRNA07	Chromosome	644274	645845	+	16S_rRNA
LL-07.rRNA08	Chromosome	646266	649185	+	23S_rRNA
LL-07.rRNA09	Chromosome	649285	649396	+	5S_rRNA
LL-07.rRNA10	Chromosome	1148891	1148780	-	5S_rRNA
LL-07.rRNA11	Chromosome	1151910	1148991	-	23S_rRNA
LL-07.rRNA12	Chromosome	1153713	1152142	-	16S_rRNA
LL-07.rRNA13	Chromosome	1440589	1440478	-	5S_rRNA
LL-07.rRNA14	Chromosome	1443608	1440689	-	23S_rRNA
LL-07.rRNA15	Chromosome	1445600	1444029	-	16S_rRNA

Supplementary Table 3 Prediction of sRNA in *P. pentosaceus* LL-07

sRNA ID	Location	Startbp	Endbp	sRNA Lengthbp	Strand	Rfam ID	Score
LL-07.sRNA01	Chromosome	133913	134093	181	+	RF00168	104.3
LL-07.sRNA02	Chromosome	341499	341388	112	-	RF00059	57.6
LL-07.sRNA03	Chromosome	364820	364947	128	+	RF02840	90.4
LL-07.sRNA04	Chromosome	528421	528487	67	+	RF01734	38.8
LL-07.sRNA05	Chromosome	579842	580081	240	+	RF00230	114.8
LL-07.sRNA06	Chromosome	589593	589698	106	+	RF00059	68.1
LL-07.sRNA07	Chromosome	654097	654177	81	+	RF01767	60.2
LL-07.sRNA08	Chromosome	658224	658428	205	+	RF00230	104.2
LL-07.sRNA09	Chromosome	714084	714211	128	+	RF00558	65.5
LL-07.sRNA10	Chromosome	755115	755182	68	+	RF00559	53.9
LL-07.sRNA11	Chromosome	914528	914900	373	+	RF00011	319.9
LL-07.sRNA12	Chromosome	1118582	1118368	215	-	RF00230	114.1
LL-07.sRNA13	Chromosome	1216025	1215795	231	-	RF00230	111.5
LL-07.sRNA14	Chromosome	1222550	1222339	212	-	RF00230	94.7
LL-07.sRNA15	Chromosome	1241476	1241234	243	-	RF00230	96.7
LL-07.sRNA16	Chromosome	1313944	1313884	61	-	RF00555	48.7
LL-07.sRNA17	Chromosome	1317350	1317294	57	-	RF01708	37.3
LL-07.sRNA18	Chromosome	1352288	1352111	178	-	RF00168	95.7
LL-07.sRNA19	Chromosome	1357962	1357578	385	-	RF01766	86.2
LL-07.sRNA20	Chromosome	1362688	1362783	96	+	RF01831	82.6
LL-07.sRNA21	Chromosome	1380157	1380406	250	+	RF00230	96.9
LL-07.sRNA22	Chromosome	1393715	1393629	87	-	RF00169	59.1
LL-07.sRNA23	Chromosome	1399757	1399631	127	-	RF00557	58.1
LL-07.sRNA24	Chromosome	1500055	1499931	125	-	RF00050	97.1
LL-07.sRNA25	Chromosome	1731784	1731652	133	-	RF00050	113.2
LL-07.sRNA26	Chromosome	1750605	1750702	98	+	RF00167	61.8

Supplementary Table 4 Carbohydrate metabolism in *P. pentosaceus* LL-07

Gene ID	KO ID	KO Name	KO Description
gene0023	K02761	*celB*	cellobiose PTS system EIIC component
gene0220	K02761	*celB*	cellobiose PTS system EIIC component
gene1716	K02761	*celB*	cellobiose PTS system EIIC component
gene0025	K23257	*yvgN*	methylglyoxal/glyoxal reductase [EC:1.1.1.283 1.1.1.-]
gene0060	K00034	*gdh*	glucose 1-dehydrogenase [EC:1.1.1.47]
gene0109	K01223	*bglA*	6-phospho-beta-glucosidase [EC:3.2.1.86]
gene0268	K01223	*bglA*	6-phospho-beta-glucosidase [EC:3.2.1.86]
gene1717	K01223	*bglA*	6-phospho-beta-glucosidase [EC:3.2.1.86]
gene0116	K00849	*galK*	galactokinase [EC:2.7.1.6]
gene0195	K00849	*galK*	galactokinase [EC:2.7.1.6]
gene0141	K00882	*fruK*	1-phosphofructokinase [EC:2.7.1.56]
gene1119	K00882	*fruK*	1-phosphofructokinase [EC:2.7.1.56]
gene0143	K01635	*lacD*	tagatose 1,6-diphosphate aldolase [EC:4.1.2.40]
gene0144	K04041	-	fructose-1,6-bisphosphatase III [EC:3.1.3.11]
gene0152	K01788	*nanE*	N-acylglucosamine-6-phosphate 2-epimerase [EC:5.1.3.9]
gene0160	K03077	*araD*	L-ribulose-5-phosphate 4-epimerase [EC:5.1.3.4]
gene0161	K01804	*araA*	L-arabinose isomerase [EC:5.3.1.4]
gene0170	K00848	*rhaB*	rhamnulokinase [EC:2.7.1.5]
gene0172	K01813	*rhaA*	L-rhamnose isomerase [EC:5.3.1.14]
gene0173	K01629	*rhaD*	rhamnulose-1-phosphate aldolase [EC:4.1.2.19]
gene0182	K01805	*xylA*	xylose isomerase [EC:5.3.1.5]
gene0183	K00854	*xylB*	xylulokinase [EC:2.7.1.17]
gene0191	K01785	*galM*	aldose 1-epimerase [EC:5.1.3.3]
gene0210	K01785	*galM*	aldose 1-epimerase [EC:5.1.3.3]
gene1010	K01785	*galM*	aldose 1-epimerase [EC:5.1.3.3]
gene0193	K01190	*lacZ*	beta-galactosidase [EC:3.2.1.23]
gene0194	K01190	*lacZ*	beta-galactosidase [EC:3.2.1.23]
gene0196	K01784	*galE*	UDP-glucose 4-epimerase [EC:5.1.3.2]
gene1424	K01784	*galE*	UDP-glucose 4-epimerase [EC:5.1.3.2]
gene0197	K00965	*galT*	UDPglucose--hexose-1-phosphate uridylyltransferase [EC:2.7.7.12]
gene0198	K01679	*fumC*	fumarate hydratase, class II [EC:4.2.1.2]
gene0207	K00016	*ldh*	L-lactate dehydrogenase [EC:1.1.1.27]
gene1503	K00016	*ldh*	L-lactate dehydrogenase [EC:1.1.1.27]
gene0235	K01958	*pyc*	pyruvate carboxylase [EC:6.4.1.1]
gene0261	K00086	*dhaT*	1,3-propanediol dehydrogenase [EC:1.1.1.202]
gene0266	K02760	*celA*	cellobiose PTS system EIIB component [EC:2.7.1.196 2.7.1.205]
gene0267	K02759	*celC*	cellobiose PTS system EIIA component [EC:2.7.1.196 2.7.1.205]
gene0271	K03366	*butA*	meso-butanediol dehydrogenase / (S,S)-butanediol dehydrogenase / diacetyl reductase [EC:1.1.1.- 1.1.1.76 1.1.1.304]
gene0278	K04042	*glmU*	bifunctional UDP-N-acetylglucosamine pyrophosphorylase / Glucosamine-1-phosphate N-acetyltransferase [EC:2.7.7.23 2.3.1.157]
gene0280	K00948	*prsA*	ribose-phosphate pyrophosphokinase [EC:2.7.6.1]
gene1122	K00948	*prsA*	ribose-phosphate pyrophosphokinase [EC:2.7.6.1]
gene0303	K00244	*frdA*	fumarate reductase flavoprotein subunit [EC:1.3.5.4]
gene0320	K02564	*nagB*	glucosamine-6-phosphate deaminase [EC:3.5.99.6]
gene0321	K00925	*ackA*	acetate kinase [EC:2.7.2.1]
gene1761	K00925	*ackA*	acetate kinase [EC:2.7.2.1]
gene0322	K00625	*pta*	phosphate acetyltransferase [EC:2.3.1.8]
gene0338	K01621	*xfp*	xylulose-5-phosphate/fructose-6-phosphate phosphoketolase [EC:4.1.2.9 4.1.2.22]
gene0390	K01652	*ilvB*	acetolactate synthase I/II/III large subunit [EC:2.2.1.6]
gene0965	K01652	*ilvB*	acetolactate synthase I/II/III large subunit [EC:2.2.1.6]

gene0391	K01575	*alsD*	acetolactate decarboxylase [EC:4.1.1.5]
gene0429	K00963	*galU*	UTP--glucose-1-phosphate uridylyltransferase [EC:2.7.7.9]
gene0431	K01835	*manB*	phosphoglucomutase [EC:5.4.2.2]
gene0444	K00134	*gapA*	glyceraldehyde 3-phosphate dehydrogenase [EC:1.2.1.12]
gene0445	K00927	*pgk*	phosphoglycerate kinase [EC:2.7.2.3]
gene0446	K01803	*tpiA*	triosephosphate isomerase (TIM) [EC:5.3.1.1]
gene0447	K01689	*eno*	enolase [EC:4.2.1.11]
gene0504	K01689	*eno*	enolase [EC:4.2.1.11]
gene0459	K00075	*murB*	UDP-N-acetylmuramate dehydrogenase [EC:1.3.1.98]
gene0462	K03431	*glmM*	phosphoglucosamine mutase [EC:5.4.2.10]
gene0463	K00820	*glmS*	glutamine---fructose-6-phosphate transaminase (isomerizing) [EC:2.6.1.16]
gene0508	K00874	*kdgK*	2-dehydro-3-deoxygluconokinase [EC:2.7.1.45]
gene0512	K00158	*poxL*	pyruvate oxidase [EC:1.2.3.3]
gene0650	K00158	*poxL*	pyruvate oxidase [EC:1.2.3.3]
gene1205	K00158	*poxL*	pyruvate oxidase [EC:1.2.3.3]
gene0588	K01854	*glf*	UDP-galactopyranose mutase [EC:5.4.99.9]
gene0608	K01791	*wecB*	UDP-N-acetylglucosamine 2-epimerase (non-hydrolysing) [EC:5.1.3.14]
gene0723	K00033	*gntZ*	6-phosphogluconate dehydrogenase [EC:1.1.1.44 1.1.1.343]
gene1314	K00033	*gntZ*	6-phosphogluconate dehydrogenase [EC:1.1.1.44 1.1.1.343]
gene0727	K01512	*acyP*	acylphosphatase [EC:3.6.1.7]
gene0742	K00845	*glk*	glucokinase [EC:2.7.1.2]
gene0749	K01915	*glnA*	glutamine synthetase [EC:6.3.1.2]
gene0781	K01783	*rpe*	ribulose-phosphate 3-epimerase [EC:5.1.3.1]
gene0806	K02160	*accB*	acetyl-CoA carboxylase biotin carboxyl carrier protein
gene0808	K01961	*accC*	acetyl-CoA carboxylase, biotin carboxylase subunit [EC:6.4.1.2 6.3.4.14]
gene0809	K01963	*accD*	acetyl-CoA carboxylase carboxyl transferase subunit beta [EC:6.4.1.2 2.1.3.15]
gene0810	K01962	*accA*	acetyl-CoA carboxylase carboxyl transferase subunit alpha [EC:6.4.1.2 2.1.3.15]
gene0875	K01641	-	hydroxymethylglutaryl-CoA synthase [EC:2.3.3.10]
gene0883	K03778	*ldhA*	D-lactate dehydrogenase [EC:1.1.1.28]
gene0969	K00865	*glxK*	glycerate 2-kinase [EC:2.7.1.165]
gene0980	K01838	*pgmB*	beta-phosphoglucomutase [EC:5.4.2.6]
gene0981	K00691	*mapA*	maltose phosphorylase [EC:2.4.1.8]
gene0996	K07106	*murQ*	N-acetylmuramic acid 6-phosphate etherase [EC:4.2.1.126]
gene1045	K00873	*pyk*	pyruvate kinase [EC:2.7.1.40]
gene1046	K00850	*pfkA*	6-phosphofructokinase 1 [EC:2.7.1.11]
gene1111	K01092	*suhB*	myo-inositol-1(or 4)-monophosphatase [EC:3.1.3.25]
gene1118	K02768	*fruB*	fructose PTS system EIIA component [EC:2.7.1.202]
gene1150	K02781	*srlB*	glucitol/sorbitol PTS system EIIA component [EC:2.7.1.198]
gene1151	K07404	*pgl*	6-phosphogluconolactonase [EC:3.1.1.31]
gene1192	K00852	*rbsK*	ribokinase [EC:2.7.1.15]
gene1641	K00852	*rbsK*	ribokinase [EC:2.7.1.15]
gene1220	K00036	*zwf*	glucose-6-phosphate 1-dehydrogenase [EC:1.1.1.49 1.1.1.363]
gene1280	K00600	*glyA*	glycine hydroxymethyltransferase [EC:2.1.2.1]
gene1287	K01809	*manA*	mannose-6-phosphate isomerase [EC:5.3.1.8]
gene1302	K01810	*pgi*	glucose-6-phosphate isomerase [EC:5.3.1.9]
gene1304	K01624	*fbaA*	fructose-bisphosphate aldolase, class II [EC:4.1.2.13]
gene1313	K00851	*gntK*	gluconokinase [EC:2.7.1.12]
gene1391	K00847	*scrK*	fructokinase [EC:2.7.1.4]
gene1455	K01807	*rpiA*	ribose 5-phosphate isomerase A [EC:5.3.1.6]
gene1459	K01834	*gpmA*	2,3-bisphosphoglycerate-dependent phosphoglycerate mutase [EC:5.4.2.11]
gene1652	K01834	*gpmA*	2,3-bisphosphoglycerate-dependent phosphoglycerate mutase [EC:5.4.2.11]

gene1462	K02769	*fruAb*	fructose PTS system EIIB component [EC:2.7.1.202]
gene1465	K02796	*manZ*	mannose PTS system EIID component
gene1699	K02796	*manZ*	mannose PTS system EIID component
gene1466	K02795	*manY*	mannose PTS system EIIC component
gene1700	K02795	*manY*	mannose PTS system EIIC component
gene1467	K02793	*manXa*	mannose PTS system EIIA component [EC:2.7.1.191]
gene1703	K02793	*manXa*	mannose PTS system EIIA component [EC:2.7.1.191]
gene1477	K01443	*nagA*	N-acetylglucosamine-6-phosphate deacetylase [EC:3.5.1.25]
gene1696	K01443	*nagA*	N-acetylglucosamine-6-phosphate deacetylase [EC:3.5.1.25]
gene1519	K00790	*murA*	UDP-N-acetylglucosamine 1-carboxyvinyltransferase [EC:2.5.1.7]
gene1560	K22212	*mleA*	malolactic enzyme [EC:4.1.1.101]
gene1636	K22373	*larA*	lactate racemase [EC:5.1.2.1]
gene1701	K02794	*manX*	mannose PTS system EIIAB component [EC:2.7.1.191]
gene1711	K00382	*pdhD*	dihydrolipoamide dehydrogenase [EC:1.8.1.4]
gene1712	K00627	*aceF*	pyruvate dehydrogenase E2 component (dihydrolipoamide acetyltransferase) [EC:2.3.1.12]
gene1713	K00162	*pdhB*	pyruvate dehydrogenase E1 component beta subunit [EC:1.2.4.1]
gene1714	K00161	*pdhA*	pyruvate dehydrogenase E1 component alpha subunit [EC:1.2.4.1]
gene1746	K01226	*treC*	trehalose-6-phosphate hydrolase [EC:3.2.1.93]

Supplementary Table 5 Gene details of prophage in *P. pentosaceus* LL-07

Prophage ID	Gene ID	Strand	Start（bp）	End（bp）	Description
	gene0817	-	805991	806881	hypothetical protein
	gene0818	-	806957	807349	ImmA/IrrE family metallo-endopeptidase
	gene0819	-	807356	807679	helix-turn-helix domain-containing protein
	gene0820	+	807822	808043	helix-turn-helix transcriptional regulator
	gene0821	+	808040	808291	hypothetical protein
	gene0822	-	808272	808463	hypothetical protein
Ph01	*gene0823*	+	808527	808769	hypothetical protein
	gene0824	+	808843	809301	helix-turn-helix domain-containing protein
	gene0825	+	809302	809577	helix-turn-helix domain-containing protein
	gene0826	+	809591	809722	hypothetical protein
	gene0827	+	809815	810096	hypothetical protein
	gene0828	+	810089	810940	recombinase RecT
	gene0829	+	810900	811727	PD-(D/E)XK nuclease-like domain-containing protein
	gene0837	+	815195	815545	DUF1642 domain-containing protein
	gene0838	+	815538	815735	hypothetical protein
	gene0839	+	815779	816069	hypothetical protein
	gene0840	+	816137	816442	hypothetical protein
	gene0841	+	816445	816639	hypothetical protein
	gene0842	+	816642	816842	hypothetical protein
	gene0843	+	816839	817012	hypothetical protein
	gene0844	+	817082	817522	hypothetical protein
	gene0846	+	817847	819043	hypothetical protein
	gene0847	+	819236	819805	terminase small subunit
	gene0848	+	819802	821175	PBSX family phage terminase large subunit
	gene0849	+	821229	822725	phage portal protein
	gene0850	+	822722	823855	capsid protein
	gene0851	+	823955	824509	phage scaffolding protein
	gene0852	+	824522	825412	hypothetical protein
	gene0853	+	825490	825957	Ig-like domain-containing protein
	gene0854	+	825969	826385	hypothetical protein
Ph02	*gene0855*	+	826382	826729	capsid protein
	gene0856	+	826729	827079	minor capsid protein
	gene0857	+	827066	827461	minor capsid protein
	gene0858	+	827465	828010	hypothetical protein
	gene0859	+	828084	828521	hypothetical protein
	gene0860	+	828528	829160	Gp15 family bacteriophage protein
	gene0861	+	829164	834437	tape measure protein
	gene0862	+	834490	835287	phage tail family protein
	gene0863	+	835296	836432	phage tail protein
	gene0864	+	836422	836748	hypothetical protein
	gene0865	+	836738	837013	hypothetical protein
	gene0866	+	837013	838833	metallophosphoesterase
	gene0867	+	838846	839601	hypothetical protein
	gene0868	+	839612	839932	DUF2977 domain-containing protein
	gene0869	+	839934	840077	XkdX family protein
	gene0870	+	840111	840245	-
	gene0871	+	840279	840680	phage holin family protein
	gene0872	+	840664	841989	Lyzozyme M1 (1,4-beta-N-acetylmuramidase)
	gene0873	+	842659	842979	acetyl-CoA carboxylase

gene0874	+	843435	844811	MULTISPECIES: amino acid permease

Supplementary Table 6 Prediction of CRISPR/Cas in *P. pentosaceus* LL-07

Element	Start（bp）	End（bp）	DR Length (bp)	Orientation
Cas3-1	434,183	435,502	-	+
Cas3-2	1,188,788	1,190,149	-	-
Cas3-3	1,466,831	1,468,411	-	-
CRISPR-1	649,410	649,515	24	-
CRISPR-2	1,686,878	1,686,990	29	+

Supplementary Table 7 Prediction of Transposon in *P. pentosaceus* LL-07

Transposon ID	Location	Start	End	Transposon families	Match Start	Match End	Evalue
chain00001	Chromosome	308858	309301	LINE	913	1033	1.00E-08
chain00002	Chromosome	767731	767937	helitronORF	859	924	1.00E-06
chain00003	Chromosome	1519704	1520369	LINE	6	205	4.00E-06

Supplementary Table 8 Detailed table of virulence gene prediction in *P. pentosaceus* LL-07

Gene ID	VFDB ID	Level 1	Level 2	Identity (%)
gene0016	VFG002176(gb|AAM75252)	Offensive virulence factors	Toxin	28.20
gene0019	VFG000344(gb|NP_438273)	Nonspecific virulence factor	Iron uptake system	30.60
gene0026	VFG038840(gb|YP_008043465)	Offensive virulence factors	Adherence	28.70
gene0049	VFG001306(gb|NP_644948)	Defensive virulence factors	Antiphagocytosis	35.70
gene0053	VFG010862(gb|YP_094891)	Nonspecific virulence factor	Iron uptake system	29.60
gene0060	VFG005767(gb|NP_687682)	Offensive virulence factors	Toxin	34.60
gene0068	VFG000574(gb|NP_462662)	Nonspecific virulence factor	Magnesium uptake system	25.70
gene0072	VFG000574(gb|NP_462662)	Nonspecific virulence factor	Magnesium uptake system	29.80
gene0080	VFG001341(gb|NP_688173)	Defensive virulence factors;Defensive virulence factors	Antiphagocytosis;Serum resistance	33.90
gene0108	VFG001285(gb|NP_647403)	Offensive virulence factors	Adherence	31.70
gene0146	VFG038840(gb|YP_008043465)	Offensive virulence factors	Adherence	29.10
gene0150	VFG000575(gb|NP_462663)	Nonspecific virulence factor	Magnesium uptake system	40.90
gene0190	VFG002197(gb|NP_814691)	-	-	25.60
gene0196	VFG002361(gb|YP_001007253)	-	-	39.30
gene0200	VFG041304(gb|YP_095978)	Offensive virulence factors	Secretion system	39.60
gene0205	VFG013260(gb|NP_438427)	-	-	27.60
gene0214	VFG013496(gb|NP_439840)	-	-	22.60
gene0232	VFG000344(gb|NP_438273)	Nonspecific virulence factor	Iron uptake system	35.10
gene0239	VFG002548(gb|YP_109384)	-	Antiphagocytosis	33.00
gene0262	VFG001267(gb|NP_252913)	Nonspecific virulence factor	Iron uptake system	27.40
gene0271	VFG005767(gb|NP_687682)	Offensive virulence factors	Toxin	34.80
gene0275	VFG002176(gb|AAM75252)	Offensive virulence factors	Toxin	28.00
gene0278	VFG047256(gb|YP_169432.1)	-	-	41.60
gene0300	VFG047726(gb|YP_170570.1)	-	-	41.20
gene0301	VFG047708(gb|YP_170571.1)	-	-	46.80
gene0307	VFG001285(gb|NP_647403)	Offensive virulence factors	Adherence	27.60
gene0316	VFG001206(gb|NP_273675)	Nonspecific virulence factor	Iron uptake system	37.80
gene0331	VFG040705(gb|YP_067664)	-	-	23.00
gene0362	VFG002176(gb|AAM75252)	Offensive virulence factors	Toxin	26.60
gene0374	VFG000344(gb|NP_438273)	Nonspecific virulence factor	Iron uptake system	28.90
gene0375	VFG001983(gb|NP_282073)	Offensive virulence factors	Adherence	31.30
gene0384	VFG000344(gb|NP_438273)	Nonspecific virulence factor	Iron uptake system	31.80
gene0385	VFG007023(gb|NP_231091)	Offensive virulence factors	Toxin	32.60
gene0397	VFG000574(gb|NP_462662)	Nonspecific virulence factor	Magnesium uptake system	28.10
gene0407	VFG001855(gb|YP_094724)	Offensive virulence factors	Adherence	56.40
gene0416	VFG038219(gb|YP_001845364)	Regulation of virulence-associated genes	Regulation	38.80
gene0417	VFG009810(gb|NP_215272)	Regulation of virulence-associated genes	Regulation	31.90
gene0421	VFG007023(gb|NP_231091)	Offensive virulence factors	Toxin	32.70
gene0422	VFG000344(gb|NP_438273)	Nonspecific virulence factor	Iron uptake system	30.10
gene0429	VFG000964(gb|NP_270109)	Defensive virulence factors;Offensive virulence factors	Antiphagocytosis;Adherence	69.90
gene0431	VFG013327(gb|NP_438900)	-	-	31.50
gene0440	VFG000077(gb|NP_465991)	Defensive virulence factors	Stress protein	71.10
gene0442	VFG043396(gb|YP_002344093)	-	-	27.50
gene0459	VFG002184(gb|NP_816135)	Defensive virulence factors	Antiphagocytosis	39.00
gene0462	VFG013327(gb|NP_438900)	-	-	22.90
gene0471	VFG037386(gb|YP_001847242)	Nonspecific virulence factor	Iron uptake system	26.30
gene0488	VFG005776(gb|NP_687685)	Offensive virulence factors	Toxin	29.90
gene0493	VFG049114(gb|YP_002918199.1)	-	-	37.10

gene0525	VFG043504(gb|NP_357669)	Offensive virulence factors	Adherence	48.60
gene0526	VFG001386(gb|NP_215271)	Regulation of virulence-associated genes	Regulation	36.40
gene0527	VFG009810(gb|NP_215272)	Regulation of virulence-associated genes	Regulation	24.90
gene0528	VFG037100(gb|NP_273110)	Defensive virulence factors	Stress protein	35.50
gene0558	VFG000329(gb|NP_439689)	-	-	28.50
gene0560	VFG038840(gb|YP_008043465)	Offensive virulence factors	Adherence	26.00
gene0578	VFG001297(gb|NP_644939)	Defensive virulence factors	Antiphagocytosis	33.10
gene0579	VFG001298(gb|NP_644940)	Defensive virulence factors	Antiphagocytosis	44.70
gene0580	VFG001299(gb|NP_644941)	Defensive virulence factors	Antiphagocytosis	35.70
gene0581	VFG013365(gb|NP_439033)	-	-	43.90
gene0582	VFG049073(gb|YP_002920346.1)	Defensive virulence factors	Serum resistance	31.30
gene0583	VFG002188(gb|NP_816139)	Defensive virulence factors	Antiphagocytosis	33.60
gene0584	VFG001341(gb|NP_688173)	Defensive virulence factors;Defensive virulence factors	Antiphagocytosis;Serum resistance	34.20
gene0588	VFG002182(gb|NP_816133)	Defensive virulence factors	Antiphagocytosis	60.90
gene0590	VFG046732(gb|YP_169804.1)	-	-	28.10
gene0608	VFG001373(gb|NP_344890)	Defensive virulence factors	Antiphagocytosis	58.90
gene0618	VFG000080(gb|NP_464522)	Defensive virulence factors	Stress protein	59.90
gene0669	VFG015551(gb|NP_248741)	-	-	29.40
gene0687	VFG032878(gb|NP_465743)	-	-	36.90
gene0690	VFG000344(gb|NP_438273)	Nonspecific virulence factor	Iron uptake system	26.90
gene0695	VFG002409(gb|NP_645079)	Offensive virulence factors	Secretion system	29.40
gene0705	VFG000120(gb|NP_253949)	Defensive virulence factors;Defensive virulence factors	Antiphagocytosis;Serum resistance	29.10
gene0706	VFG000119(gb|NP_253948)	Defensive virulence factors;Defensive virulence factors	Antiphagocytosis;Serum resistance	31.40
gene0723	VFG048830(gb|YP_002920353.1)	Defensive virulence factors	Antiphagocytosis	67.90
gene0724	VFG038219(gb|YP_001845364)	Regulation of virulence-associated genes	Regulation	40.10
gene0725	VFG009810(gb|NP_215272)	Regulation of virulence-associated genes	Regulation	27.80
gene0770	VFG037118(gb|NP_273782)	Defensive virulence factors	Stress protein	32.30
gene0779	VFG002060(gb|NP_248764)	Offensive virulence factors	Secretion system	36.30
gene0781	VFG046610(gb|YP_169796.1)	-	-	41.90
gene0788	VFG011430(gb|NP_540392)	-	-	40.30
gene0800	VFG011399(gb|NP_539749)	-	-	41.00
gene0803	VFG049155(gb|YP_006635481.1)	Offensive virulence factors	Toxin	27.80
gene0804	VFG038840(gb|YP_008043465)	Offensive virulence factors	Adherence	43.40
gene0888	VFG002190(gb|NP_816141)	Defensive virulence factors	Antiphagocytosis	61.90
gene0889	VFG002189(gb|NP_816140)	Defensive virulence factors	Antiphagocytosis	50.00
gene0890	VFG015000(gb|NP_252339)	Defensive virulence factors	Antiphagocytosis	33.20
gene0893	VFG039491(gb|NP_820417)	Offensive virulence factors	Secretion system	40.60
gene0909	VFG038839(gb|YP_858598)	Offensive virulence factors	Adherence	36.00
gene0913	VFG000969(gb|NP_269989)	Nonspecific virulence factor	Exoenzyme	32.50
gene0928	VFG047055(gb|YP_170389.1)	-	-	39.00
gene0949	VFG002161(gb|NP_465369)	-	-	40.80
gene0952	VFG047726(gb|YP_170570.1)	-	-	34.30
gene0953	VFG047708(gb|YP_170571.1)	-	-	31.10
gene0954	VFG002160(gb|NP_465354)	Offensive virulence factors	Adherence	45.30
gene0967	VFG037100(gb|NP_273110)	Defensive virulence factors	Stress protein	38.20
gene0968	VFG037100(gb|NP_273110)	Defensive virulence factors	Stress protein	63.50
gene0983	VFG002197(gb|NP_814691)	-	-	34.70
gene0987	VFG000080(gb|NP_464522)	Defensive virulence factors	Stress protein	46.00

gene1018	VFG001206(gb|NP_273675)	Nonspecific virulence factor	Iron uptake system	32.90
gene1022	VFG000344(gb|NP_438273)	Nonspecific virulence factor	Iron uptake system	25.70
gene1049	VFG002076(gb|NP_248780)	Offensive virulence factors	Secretion system	39.20
gene1061	VFG045566(gb|YP_096368)	Offensive virulence factors	Secretion system	42.20
gene1065	VFG002188(gb|NP_816139)	Defensive virulence factors	Antiphagocytosis	41.10
gene1073	VFG001826(gb|NP_217099)	Regulation of virulence-associated genes	Regulation	40.30
gene1089	VFG001206(gb|NP_273675)	Nonspecific virulence factor	Iron uptake system	35.80
gene1096	VFG046465(gb|YP_169203.1)	Offensive virulence factors	Adherence	69.20
gene1106	VFG000320(gb|NP_208266)	Offensive virulence factors	Adherence	36.20
gene1147	VFG002409(gb|NP_645079)	Offensive virulence factors	Secretion system	26.60
gene1191	VFG001876(gb|YP_095349)	Offensive virulence factors	Secretion system	31.60
gene1193	VFG039536(gb|NP_820549)	Offensive virulence factors	Secretion system	46.50
gene1203	VFG002197(gb|NP_814691)	-	-	28.10
gene1209	VFG013265(gb|NP_438428)	-	-	44.80
gene1233	VFG038840(gb|YP_008043465)	Offensive virulence factors	Adherence	30.20
gene1238	VFG000344(gb|NP_438273)	Nonspecific virulence factor	Iron uptake system	39.20
gene1264	VFG005776(gb|NP_687685)	Offensive virulence factors	Toxin	34.30
gene1270	VFG001254(gb|NP_249795)	Offensive virulence factors	Adherence	32.70
gene1272	VFG007158(gb|NP_798047)	Offensive virulence factors	Secretion system	29.90
gene1278	VFG000344(gb|NP_438273)	Nonspecific virulence factor	Iron uptake system	33.80
gene1289	VFG013248(gb|NP_438233)	-	-	32.90
gene1290	VFG013248(gb|NP_438233)	-	-	27.70
gene1305	VFG009810(gb|NP_215272)	Regulation of virulence-associated genes	Regulation	28.00
gene1306	VFG038219(gb|YP_001845364)	Regulation of virulence-associated genes	Regulation	37.80
gene1314	VFG048830(gb|YP_002920353.1)	Defensive virulence factors	Antiphagocytosis	35.30
gene1339	VFG001206(gb|NP_273675)	Nonspecific virulence factor	Iron uptake system	30.90
gene1340	VFG037386(gb|YP_001847242)	Nonspecific virulence factor	Iron uptake system	29.20
gene1377	VFG000079(gb|NP_463763)	Defensive virulence factors	Stress protein	55.80
gene1407	VFG047582(gb|YP_169891.1)	-	-	32.40
gene1410	VFG047564(gb|YP_169890.1)	-	-	38.80
gene1411	VFG015551(gb|NP_248741)	-	-	29.20
gene1424	VFG002361(gb|YP_001007253)	-	-	44.80
gene1463	VFG002085(gb|NP_232518)	Offensive virulence factors	Secretion system	32.00
gene1472	VFG000574(gb|NP_462662)	Nonspecific virulence factor	Magnesium uptake system	29.50
gene1518	VFG000812(gb|NP_290268)	Offensive virulence factors	Secretion system	29.70
gene1538	VFG000130(gb|NP_252238)	Defensive virulence factors;Defensive virulence factors	Antiphagocytosis;Serum resistance	33.30
gene1539	VFG016058(gb|NP_251114)	Nonspecific virulence factor	Iron uptake system	28.50
gene1546	VFG002188(gb|NP_816139)	Defensive virulence factors	Antiphagocytosis	44.90
gene1549	VFG002181(gb|NP_816132)	Defensive virulence factors	Antiphagocytosis	49.90
gene1550	VFG002180(gb|NP_816131)	Defensive virulence factors	Antiphagocytosis	41.10
gene1551	VFG002188(gb|NP_816139)	Defensive virulence factors	Antiphagocytosis	41.90
gene1553	VFG000670(gb|NP_706258)	-	-	41.10
gene1570	VFG047654(gb|YP_169518.1)	-	-	22.30
gene1571	VFG047654(gb|YP_169518.1)	-	-	22.50
gene1583	VFG001306(gb|NP_644948)	Defensive virulence factors	Antiphagocytosis	51.60
gene1588	VFG000574(gb|NP_462662)	Nonspecific virulence factor	Magnesium uptake system	28.60
gene1592	VFG002176(gb|AAM75252)	Offensive virulence factors	Toxin	25.30
gene1593	VFG001206(gb|NP_273675)	Nonspecific virulence factor	Iron uptake system	32.70
gene1594	VFG000574(gb|NP_462662)	Nonspecific virulence factor	Magnesium uptake system	30.20
gene1598	VFG000344(gb|NP_438273)	Nonspecific virulence factor	Iron uptake system	33.10

gene1601	VFG013248(gb|NP_438233)	-	-	27.50
gene1624	VFG005776(gb|NP_687685)	Offensive virulence factors	Toxin	28.90
gene1628	VFG044083(gb|NP_250948)	Nonspecific virulence factor	Iron uptake system	26.40
gene1642	VFG002197(gb|NP_814691)	-	-	28.50
gene1658	VFG009810(gb|NP_215272)	Regulation of virulence-associated genes	Regulation	28.30
gene1659	VFG038219(gb|YP_001845364)	Regulation of virulence-associated genes	Regulation	38.80
gene1675	VFG000119(gb|NP_253948)	Defensive virulence factors;Defensive virulence factors	Antiphagocytosis;Serum resistance	32.40
gene1678	VFG001206(gb|NP_273675)	Nonspecific virulence factor	Iron uptake system	29.30
gene1685	VFG005776(gb|NP_687685)	Offensive virulence factors	Toxin	36.10
gene1715	VFG002158(gb|NP_464456)	-	-	37.30
gene1721	VFG002437(gb|YP_108306)	Offensive virulence factors	Adherence	24.70
gene1732	VFG014950(gb|NP_249457)	Defensive virulence factors	Antiphagocytosis	38.80
gene1736	VFG009810(gb|NP_215272)	Regulation of virulence-associated genes	Regulation	32.40
gene1737	VFG038219(gb|YP_001845364)	Regulation of virulence-associated genes	Regulation	40.90
gene1740	VFG044083(gb|NP_250948)	Nonspecific virulence factor	Iron uptake system	26.50
gene1748	VFG002197(gb|NP_814691)	-	-	24.60
gene1752	VFG001206(gb|NP_273675)	Nonspecific virulence factor	Iron uptake system	33.20

Supplementary Table 9 Statistical table of prediction and classification of antibiotic resistance genes in *P. pentosaceus* LL-07

Drug Class	Gene No.	Drug Class	Gene No.
Macrolide antibiotic	26	Peptide antibiotic	5
Tetracycline antibiotic	16	Penam	5
Fluoroquinolone antibiotic	10	Mupirocin	4
Lincosamide antibiotic	10	Diaminopyrimidine antibiotic	3
Phenicol antibiotic	9	Nitroimidazole antibiotic	3
Streptogramin antibiotic	8	Cephalosporin	2
Oxazolidinone antibiotic	7	Isoniazid	2
Pleuromutilin antibiotic	6	Fosfomycin	2
Aminoglycoside antibiotic	6	Carbapenem	1
Glycopeptide antibiotic	6	Monobactam	1
Aminocoumarin antibiotic	6	Penem	1
Acridine dye	6	Cephamycin	1
Rifamycin antibiotic	5		

Supplementary Table 10 The probiotic-related genes in *P. pentosaceus* LL-07

Characterization	Gene ID	Length (bp)	Gene Description
Bile tolerance	*gene0057*	1,026	Choloylglycine hydrolase family protein
Acid tolerance	*gene1269*	420	F0F1 ATP synthase subunit epsilon
	gene1270	1,410	F0F1 ATP synthase subunit beta
	gene1271	921	F0F1 ATP synthase subunit gamma
	gene1272	1,518	F0F1 ATP synthase subunit alpha
	gene1273	543	F0F1 ATP synthase subunit delta
	gene1274	522	F0F1 ATP synthase subunit B
	gene1275	213	F0F1 ATP synthase subunit C
	gene1276	717	F0F1 ATP synthase subunit A
Universal stress resistance	*gene0046*	432	Universal stress protein
	gene0258	474	Universal stress protein
	gene0259	450	Universal stress protein
	gene0340	453	Universal stress protein
	gene0660	453	Universal stress protein
	gene1253	456	Universal stress protein
Adhesion	*gene0406*	284	Co chaperone GroES
	gene0407	1,619	Chaperonin GroEL
	gene0903	1,859	Molecular chaperone DnaK
	gene1002	761	LPXTG cell wall anchor domain containing protein
	gene1003	2,435	LPXTG cell wall anchor domain containing protein
	gene1096	1,187	Elongation factor Tu
	gene1191	962	Flp pilus assembly complex ATPase component TadA
	gene1267	257	Membrane protein insertion efficiency factor YidD
	gene1515	689	Class A sortase
	gene1783	839	Membrane protein insertase YidC
Antioxidant	*gene0132*	764	4-hydroxy-tetrahydrodipicolinate reductase
	gene0133	899	4-hydroxy-tetrahydrodipicolinate reductase
	gene1049	2,591	ATP-dependent chaperone ClpB
	gene0618	2,219	ATP-dependent Clp protease ATP-binding subunit
	gene1377	2,465	ATP-dependent Clp protease ATP-binding subunit
	gene1094	1,256	ATP-dependent Clp protease ATP-binding subunit ClpX
	gene0045	1,601	Divalent metal cation transporter MntH
	gene1436	230	Glutaredoxin-like protein NrdH
	gene0027	794	NAD(P)H-dependent oxidoreductase
	gene0961	668	NAD(P)H-dependent oxidoreductase
	gene1001	599	NAD(P)H-dependent oxidoreductase
	gene1300	545	NAD(P)H-dependent oxidoreductase
	gene1607	683	NAD(P)H-dependent oxidoreductase
	gene1600	1,040	Oxidoreductase
	gene0968	443	Peptide-methionine (R)-S-oxide reductase MsrB
	gene0528	512	Peptide-methionine (S)-S-oxide reductase MsrA
	gene0967	515	Peptide-methionine (S)-S-oxide reductase MsrA
	gene0026	794	SDR family oxidoreductase
	gene0050	749	SDR family oxidoreductase
	gene0060	785	SDR family oxidoreductase

	gene0146	893	SDR family oxidoreductase
	gene0222	638	SDR family oxidoreductase
	gene0497	743	SDR family oxidoreductase
	gene0524	107	SDR family oxidoreductase
	gene1233	725	SDR family oxidoreductase
	gene1650	632	SDR family oxidoreductase
	gene1710	851	SDR family oxidoreductase
	gene0358	329	Thioredoxin
	gene1211	311	Thioredoxin
	gene0430	926	Thioredoxin-disulfide reductase
	gene0440	593	Thioredoxin-disulfide reductase
Folic acid synthesis	*Gene1243*	1,275	Bifunctional folylpolyglutamate synthase/dihydrofolate synthase
